# Haemonchosis: A Challenging Parasitic Infection of Sheep and Goats

**DOI:** 10.3390/ani11020363

**Published:** 2021-02-01

**Authors:** Konstantinos V. Arsenopoulos, George C. Fthenakis, Eleni I. Katsarou, Elias Papadopoulos

**Affiliations:** 1 Laboratory of Parasitology and Parasitic Diseases, School of Veterinary Medicine, Faculty of Health Sciences, Aristotle University of Thessaloniki, 54124 Thessaloniki, Greece; arsenopo@vet.auth.gr; 2 Veterinary Faculty, University of Thessaly, 43100 Karditsa, Greece; gcf@vet.uth.gr (G.C.F.); elekatsarou@vet.uth.gr (E.I.K.)

**Keywords:** abomasum, anthelmintic resistance, diagnosis, goat, *Haemonchus*, health management, prevention, sheep, treatment

## Abstract

**Simple Summary:**

The paper reviews the challenges regarding haemonchosis, a common parasitic infection of sheep and goats, caused by *Haemonchus* parasites. The disease affects the health and welfare of sheep and goats and reduces their productivity, and thus, currently, there are various concerns regarding the disease. These refer to (a) the varying prevalence of the infection around the world as influenced by differing climatic conditions and environmental factors, (b) the increased resistance of the causative parasites to the available antiparasitic drugs, (c) the difficulties present regarding the accurate diagnosis of the disease and (d) the effective control of the infection.

**Abstract:**

The paper reviews the challenges about haemonchosis—a significant and common parasitic infection of small ruminants. *Haemonchus contortus* is a highly pathogenic parasite that localises in the abomasum of affected animals and exerts its pathogenicity by blood-sucking activity, adversely affecting the health and productivity of animals. The first challenge is the uneven distribution of the infection globally, this being more prevalent in tropical and subtropical and warm temperate and summer rainfall regions than in cool and cold temperate and arid regions; hence, this leads in differences in the approaches required for its control. Another challenge is the widespread presence of *Haemonchus* strains resistant to the various anthelmintics available: Benzimidazoles, imidazothiazoles, macrocyclic lactones, closantel and monepantel, which makes the control of the infection difficult. The third challenge refers to the difficulty of diagnosing the disease, given that field evidence can provide suspicion about the infection, which needs to be subsequently confirmed by laboratory tests through parasitological or molecular techniques. The final challenge relates to the difficulties in the control of the infection and the necessity to use pharmaceutical products cautiously and with a planned approach, to avoid further development of anthelmintic resistance, also given that use of a recently licenced vaccine is not widespread. In conclusion, at the moment, we should be concerned, but not worried, about this infection, and apply correctly the appropriate health management plans.

## 1. Background: The *Haemonchus* Parasites

The genus *Haemonchus* spp. was first described in 1803 by Karl Rudolphi [1] and includes blood-sucking nematode helminths. Taxonomically, the genus belongs to the superfamily Trichostrongylidae. Along with *Teladorsagia* spp., *Haemonchus* parasites are the most pathogenic nematode helminths of small ruminants. *Haemonchus* spp. can infect all species of ruminants [1]. The parasites express their pathogenicity through their blood-feeding activity [2].

The genus includes at least 12 species (Appendix A). *Haemonchus contortus* and *H. placei* are the most widespread species of the genus. *H. contortus* infects mainly grazing small ruminants, while *H. placei* has been recovered from large ruminants (i.e., cattle). *H. contortus* is globally distributed and parasitises the abomasum of the above animals, as well as of other, non-domesticated ruminants. Less common species in the genus include *H. similis*, which infects mainly cattle [3], and *H. longistipes*, which has been recovered from camels [4]. Moreover, epidemiological studies in various countries have also revealed the presence of hybrid parasites [5,6].

*Haemonchus* spp. has a direct life cycle. It is transmitted horizontally through grazing on natural pasturelands by third stage (L_3_) larvae. It has a two-phase life cycle, a free-living and a parasitic within the abomasum of the host. The eggs reach the ground through faeces of the parasitised ruminants, thus infecting herbage. Then, the eggs evolve to first stage larvae (L_1_), continuing to second stage larvae (L_2_). The third stage larvae (L_3_), which is the infective form of this parasite, is the next evolutionary stage. After L_3_ larvae have been uptaken by a ruminant, they migrate to the predilection site, i.e., the abomasum, where they become adult nematode parasites, expressing their blood-sucking activity [2,7].

*H. contortus* infection leads to anaemia, primarily due to the blood-sucking activity, and consequently, potentially occasional death of the infected animals. An alternate pathogenetic mechanism was presented by Fetterer and Rhoads [8], who described that the helminth produced a haemolytic factor that caused distinct morphological changes on the surface of erythrocytes of affected sheep. Nevertheless, the primary consequence of the infection is the significant reduction in the production of infected animals, which includes a decrease in the growth of young animals, a decrease in milk production of lactating animals and in fibre production [9]. In the end, all these result in severe economic losses for farmers [10].

The importance of this helminth is highlighted by the fact that annual treatments for *H. contortus* alone have been estimated to amount to approximately 26 million USD in Kenya, 46 million USD in the Republic of South Africa and 103 million USD in India [11].

Climatic conditions have a significant impact on the development of the infection. A humid and warm climate is the optimum combination for *Haemonchus* spp. and infection of ruminants. Many researchers have investigated the environmental conditions, in which the development process of the first three larval stages (eggs to L_3_ larvae) is successful. According to Gordon [12], the lowest environmental limit referred to a combination of monthly average 18 °C temperature and 50 mm rainfall. According to Rossanigo and Gruner [13], the highest environmental limits for developing the parasitic eggs to L_3_ larvae are 28 °C temperature and over 70% humidity. Little or no development of the parasitic eggs to L_3_ larvae would occur in temperatures below 9 °C [14]. Therefore, it becomes evident that the free-living stages of the helminth do not develop easily in cold environments. Therefore, it is plausible that *Haemonchus* spp. would prefer tropical and subtropical areas, where environmental conditions favour its survival.

*Haemonchus* spp. possesses a unique feature, which refers to inhibited development, termed ‘hypobiosis’ [15]. Seasonal changes, particularly temperature changes, have been proposed to be the major determinant of hypobiosis. The feature is activated when opportunities for transmission of the larvae are restricted, which is a parasitic adaptation in cold weather [16]. Hypobiosis can also occur in high temperature conditions during the dry season (i.e., low humidity conditions) [17].

The objectives of this paper are to review the current challenges about haemonchosis, a significant and common parasitic infection of small ruminants, and to discuss the relevant issues, with a view to optimising the control of the infection.

## 2. Challenge I: The Changing Face of the Infection

Epidemiological studies on haemonchosis have focused mainly on sequential observations of parasitic burdens in grazing ruminants, indicating the direct relationships between the parasite development and various environmental factors. These epidemiological studies have been based on three different approaches, which have included: (a) the use of animals as tracers, which grazed on pastures contaminated with parasite eggs and larvae at specific time-points, (b) worm counts in faecal samples from ruminants naturally infected and continuously challenged by nematodes and (c) abattoir surveys.

The main disadvantage of the epidemiological studies that emphasise on haemonchosis is that the majority of grazing ruminants were infected by varying species of nematodes. These co-existing species develop competition—affecting the numbers of worms which are counted. Many epidemiological studies regarding *H. contortus* are available in the literature, but not all define its epidemiology throughout a year, attributing this failure to the significant between-studies variation of frequency and severity of observations. Nevertheless, they provide an opportunity to highlight the effect of the season (i.e., effect of temperature-moisture) on the range of nematode populations among different environments, to applicate proper antiparasitic strategies [18]. Finally, faecal egg counts are not necessarily indicative of the actual worm burden, and, therefore, such studies should not be evaluated for epidemiological reasons. In contrast, the major advantage of worm counts from grazing animals is that they take into account the presence of larvae under hypobiosis, which consist of another way of *Haemonchus* spp. survival method during unfavourable climatic conditions.

Herebelow, the various climatic zones and the respective epidemiological features of *H. contortus* infections in sheep and goats are discussed in detail. These are summarised in Table 1.

### 2.1. Tropical and Subtropical Regions

Tropical and subtropical regions include the tropical regions of Africa and America, the Caribbean, the tropical islands of the Pacific Ocean, the southern and south-east Asia, the southern USA and the northern part of Australia. In these regions, L_3_ larvae do not survive on pasturelands for long periods, although the prevailing temperatures permit their development all year-round. Moisture plays a crucial role in larval survival, allowing little development in short periods during dry seasons. In high altitude areas, when moisture is adequate (i.e., during cooler winter periods), the period of larval survival and development enlarges.

Haemonchosis is a continuous threat in these areas, as larval populations develop constantly, and animals can be continuously challenged [19,20,21]. In areas where annual dry seasons occur (e.g., Cameroon, Ethiopia, Gambia, Ghana, Kenya, Nigeria, Senegal), L_3_ larvae reach peak numbers seasonally, depending on increased moisture, due to higher rainfalls [22,23,24,25,26,27,28,29]. Hypobiotic L_4_ larvae develop only during dry seasons [27,30], but this situation has not been reported in such areas, because L_3_ larvae were present nearly all year-round [31,32].

### 2.2. Warm Temperate and Summer Rainfall Regions

Warm temperate and summer rainfall regions include areas in southern and eastern Asia, southern Africa, eastern Australia, parts of southern USA and South America. In these areas, the combination of high environmental temperature and moisture, especially during the summer months, supports the development of L_3_ larvae. However, this development can be limited during dry seasons of the year. Both rainfalls and temperature are key factors, which permit the larval survival and development during other seasons of the year, a process that may slow down under cold climatic conditions of winter, especially in high altitude.

Haemonchosis is a significant threat for livestock during the warmer months, depending on the rainfalls [33,34]. In areas with a mild temperature during winter, the availability of L_3_ larvae occurs nearly all year compared to subtropical zones [34], while in areas suffering low winter temperatures, haemonchosis outbreaks depend on the seasonality [35]. In warm temperate and summer rainfall regions, hypobiosis varies greatly and is predominant only during the cold winter periods [36].

### 2.3. Mediterranean Climatic Regions

The Mediterranean-type climatic regions include the para-Mediterranean basin, Western Australia, the south-west Cape of South Africa and south-east Australia. These regions are characterised by hot, dry summer conditions, as well as too cold winter conditions. Under these environmental conditions, survival and development of all free-living stages of *Haemonchus* spp. are suspended. Therefore, larval populations reach their peak numbers in the autumn and spring. In the case of mild temperatures during the winter, there could be a survival of L_3_ larvae.

In Mediterranean climatic regions, the patterns of *Haemonchus* spp. infection in small ruminants is usually bi-phasic, with the highest *H. contortus* populations being developed from late autumn to early winter, as well as from late spring to early summer [37]. Nevertheless, it must be mentioned that the pattern of haemonchosis changes not only between years, but also between different locations within this zone, due to the varying frequency and persistency of rainfalls [38,39]. Hypobiosis varies, also, and this is related to the duration and intensity of the hot, dry conditions [40].

### 2.4. Cool and Cold Temperate Regions

Cool and cold temperate regions include regions of northern Europe, Scandinavia, United Kingdom, northern USA and Canada, south east-Australia and the islands of New Zealand. In these regions, the low temperature is a detrimental factor, which affects the risk of haemonchosis. To be precise, these too cold temperatures lead to cessation of larval development until the onset of milder environmental conditions.

The risk of haemonchosis is typically low under these climatic conditions, limited to the warmer months of the year [41,42]. L_3_ larvae, which have been consumed by the animals during the autumn in frigid zones, express hypobiosis, the most contributing mechanism, which permits the survival of these larval populations overwinter [43,44,45]. However, during the short summer, the high temperatures favour the rapid development of hypobiotic larvae, thus causing haemonchosis [46].

### 2.5. Arid Regions

Arid regions include mainly deserts of the southern and sub-Saharan Africa, the Middle East, as well as continental Australia, where the level of rainfalls remains low. Under such environmental conditions, lack of moisture is a crucial limitation for survival and development of larval populations, which are, thus, favoured only during the short rainfall periods.

*H. contortus* does not consist of a threat for the ruminants in arid zones, as the helminths remain in low numbers and the clinical appearance of this parasitosis is rather rare [47]. However, long rainfall periods can increase larval availability, and therefore, their parasitic burden [48]. Hypobiosis is usually of varying importance, due to the adverse environmental conditions under these environmental conditions [49]; for example, Jacquiet et al. [50], in a study of the ecology of sympatric *Haemonchus* spp. parasites of domestic ruminants in Mauritania, have reported that the parasites could survive during the long dry season in sheep and goats, mainly as fourth stage larvae (L_4_) in the mucosae. Despite the fact that the risk of *Haemonchus* spp. infection is higher in areas with irrigated pasturelands [51], and the hot climate decreases significantly the larval populations.

### 2.6. Current Situation

During the mid-2010s, there is a consensus among scientists that a geographical expansion of *H. contortus* populations (tropical and subtropical nematode) has been done especially in colder temperate climatic conditions of the northern hemisphere. Under these climatic conditions, the constantly increasing risk of haemonchosis has been attributed to the rise of the global temperature, which consists of the onset of multiple climate changes [46,52,53]. In this respect, the proportions of infection and spread of *H. contortus,* recorded in domestic and non-domestic ruminants, could be a useful tool indicating environmental changes, which can be applied to minimise their impact not only in animals’ but also in people health [54].

## 3. Challenge II: The Development of Anthelmintic Resistance

*Haemonchus* helminths have been extensively studied; many works have explored the mechanisms of resistance to the three main classes of anthelmintic drugs (benzimidazoles, imidazothiazoles, macrocyclic lactones) and have increased the relevant knowledge, although there are still points and issues that need to be elucidated. These have been studied in individual wild strains or laboratory strains of the parasite, hence, they may not fully reflect and encompass all the pathways leading to developing anthelmintic resistance in the field [55].

Here, the situation and the mechanisms for the resistance of *H. contortus* against the various anthelmintic drugs are discussed in detail. These are summarised in Table 2.

### 3.1. Resistance to Benzimidazoles

Lacey and Prichard first described, in 1986, a direct relationship between *β*-tubulin, which was the site-target of benzimidazoles and the development of benzimidazole-resistance in *H. contortus*. The same authors observed a reduction in the binding of tritium-labelled benzimidazoles to *β*-tubulin in resistant *H. contortus* compared to susceptible ones [56]. Lubega and Prichard [57], using a similar experimental approach, demonstrated that resistant *H. contortus* strains were associated with a reduction in the ability of benzimidazoles to bind on *β*-tubulin. The explanation for this reduced binding ability of benzimidazoles was elucidated by Kwa et al. [58,59], using *H. contortus* and *Caenorhabditis elegans*. These researchers [58,59] observed that the transfer of the gene encoding isotype 1 of *β*-tubulin from a benzimidazole-sensitive *H. contortus* to a benzimidazole-resistant *C. elegans* provided to the latter helminth susceptibility properties against benzimidazoles. The replacement of the amino acid tyrosine by another amino acid phenylalanine at the position 200 of the *β*-tubulin gene of *H. contortus*, after in vitro mutation, resulted in the loss of benzimidazole-resistance in this strain [58,59,60,61]. This study highlighted the important role of the amino acids tyrosine-phenylalanine at position 200 of the *β*-tubulin gene in the susceptibility or resistance of *Haemonchus* spp. against this anthelmintic class.

Other mutations in *H. contortus* isotype-1 of *β*-tubulin have also been linked to resistance against benzimidazoles. More precisely, a polymorphism in codon 167 of the gene encoding isotype 1 of *β*-tubulin of *H. contortus* has been associated with benzimidazole-resistance [62,63]. In the case of *H. contortus*, a point mutation (i.e., TTC to TAC) at codon 167 leads to a phenylalanine (Phe) to tyrosine (Tyr) substitution, which in turn leads to benzimidazole-resistance. Williamson et al. [64] reported a high frequency (40%) of this polymorphism in resistant *Haemonchus* spp., despite the fact that this polymorphism was rare at flock level. Ghisi et al. [65] reported a polymorphism at another position (position 198), which created benzimidazole-resistance properties to *H. contortus*. A point mutation (GCA to GAA) at codon 198 leads to alanine (Ala) to glutamic acid (Glu) substitution and can also lead to the development of resistance. Kotze et al. [66], after molecular detection of both polymorphisms at positions 198 and 200 in a *Haemonchus* spp. population, reported a higher frequency of the polymorphism at position 198 compared to the respective at position 200. Although polymorphisms at positions 167, 198 and 200 of the *β*-tubulin gene confer resistance to benzimidazoles in *H. contortus*, the combination of two or three polymorphisms has never been observed in the same individual parasite [67,68]. Further, Silvestre et al. [69] suggested more hypotheses regarding the origin of *β*-tubulin resistance alleles in *H. contortus* populations, using neutral microsatellite and anthelmintic resistance markers; specifically, the possibility of new alleles in isolated populations generating the recurrent mutation or the introduction of existing alleles, can be added to the possibility that the *β*-tubulin resistance alleles originate from a single mutational event. Although several studies have associated polymorphisms of isotype 2 of the *β*-tubulin gene with benzimidazole-resistance, this isotype was either absent or occurred with reduced frequency in the parasitic population [70,71,72,73,74].

### 3.2. Resistance to Imidazothiazoles

Sangster et al. [75] first-studied the possible mechanisms, which stimulated resistance of *H. contortus* to levamisole. Their study was based on the in vitro observation of the longitudinal contraction of adult *H. contortus* after the addition of acetylcholine, which indicated the relationship of imidazothiazoles with cholinergic receptors. Subsequent studies have shown that the ability of levamisole to bind on nicotinic acetylcholine receptors appeared to be reduced in imidazothiazole-resistant adult *H. contortus* compared to imidazothiazole-susceptible ones [76,77]. The above findings have shown that the resistance of this parasite to imidazothiazoles was based on changes in the acetylcholine receptors (site-target) of resistant *H. contortus*. A study, conducted by Sangster et al. [78], indicated that the mode of inheritance of imidazothiazole-resistance was polygenic (i.e., through the participation of multiple genes).

Recently, several molecular studies have elucidated the mechanisms for developing imidazothiazole-resistant *H. contortus* helminths. According to these studies, the development of imidazothiazole-resistance resulted from mutations in the genes encoding the nicotinic acetylcholine receptor subunit. More specifically, the mutated genes, *Hco-unc-63* and *Hco-acr-8*, have been detected in imidazothiazole-resistant *Haemonchus* spp., while they were absent from imidazothiazole-sensitive strains [64,79,80,81]. These genes express the production of a protein, which binds to the nicotinic acetylcholine receptors, preventing the binding of levamisole on them. Boulin et al. [82] have examined the effect of *Hco-unc-63* mutant protein of levamisole-resistant *H. contortus* on the ability of levamisole to bind on nicotinic acetylcholine receptors in susceptible parasitic strains (parasites with absence of this specific protein). The same researchers also observed that the ability of levamisole to bind on nicotinic acetylcholine receptors was reduced in parasites, which have expressed both normal (non-mutated) and mutated protein (presence of the *Hco-unc-63* gene) [82]. In other words, they concluded that only heterozygote *H. contortus* could resist levamisole. Barrere et al. [83] investigated the mechanism, through which the mutated *Hco-acr-8* gene was associated with imidazothiazole-resistance; after developing a novel DNA-based assay using this DNA marker for the detection and monitoring of levamisole resistance they examined nematode parasites of the genus *Haemonchus* from different geographical areas and identified a specific 63 base-pairs sequence in the normal gene, which expressed the normal (non-mutant) protein, while in the absence of this sequence, the mutant protein was expressed, reducing the ability of levamisole to bind to nicotinic receptors [83]. dos Santos [84] standardised a real-time PCR (qPCR) protocol for diagnosis of levamisole resistance in *H. contortus* populations, based on this polymorphism, and through work in many different field isolates, showed that levamisole resistance might be present in field populations, but is not as widespread as benzimidazole. Williamson et al. [64] and Sarai et al. [85] reported a significant decrease in the expression of the *Hco-unc-29.3* and *Hco-unc-63* genes, respectively, in imidazothiazole-resistant *H. contortus*. In addition, Sarai et al. [85] reported a decrease in the expression of all four types of the *Hco-unc-29* gene (*Hco-unc-29.1*, *-29.2*, *-29.3*, *-29.4*) in adult individuals of a resistant *H. contortus* strain. Sarai et al. [86] thoroughly examined that strain, based on its subpopulations, and observed increased levels of imidazothiazole-resistance in all subpopulations, which were associated with decreased expression of the genes encoding the nicotinic acetylcholine receptors, *Hco-unc-63a*, *-63b*, *-29.2*, *-29.4*, *-26* and *-acr-8a* [86].

The proposed mechanisms by which nematodes parasites of the genus *Haemonchus* resist the action of imidazothiazoles, are related to the hypofunction of nicotinic acetylcholine receptors [76,77]. However, none of these mechanisms have been extensively studied, and therefore, different mechanisms of resistance against imidazothiazoles cannot be ruled out. For example, Neveu et al. [79], after the examination of two imidazothiazole-resistant strains of *H. contortus*, identified the mutated *Hco-unc-63* gene in only one resistant *H. contortus* isolate. Williamson et al. [64] failed to detect the mutated *Hco-unc-63* gene in a resistant strain of this parasite. Sarai et al. [85] identified this gene in one susceptible and three resistant *H. contortus* worms. Sarai et al. [86] identified the responsible gene, *Hco-acr-8*, in imidazothiazole-resistant and -susceptible larvae, while they were absent from resistant adult *H. contortus*.

In conclusion, *Haemonchus* spp. possess the ability to develop a variety of mechanisms (multiple mutations), by which they reduce the sensitivity of nicotinic acetylcholine receptors to levamisole. As a consequence, this makes development of molecular methods for detecting anthelmintic resistance to imidazothiazoles difficult. It is, however, noteworthy that Sarai et al. [87] have raised the issue that in vitro levamisole selection pressure on larval stages of *H. contortus* gives rise to drug resistance, and target site gene expression changes specific only to the early larval stages, hence caution would be required in extrapolating larval-based laboratory selection results to field resistance. This was concluded by monitoring changes in larval and adult drug sensitivities and target site gene expression patterns after applying in vitro levamisole selection pressure to early stage larvae over nine.

### 3.3. Resistance to Macrocyclic Lactones

The mechanisms by which nematode parasites of the genus *Haemonchus* develop resistance to macrocyclic lactones, have been extensively studied by several researchers. Rohrer et al. [88] did not observe differences in the ability of ivermectin to bind to glutamate-gated chloride ion channels in susceptible or resistant *H. contortus* strains. In contrast, Blackhall et al. [89] recorded the increased frequency of an allele in the *α*-subunit of the gene encoding glutamate-gated chloride ion channels in a laboratory *H. contortus* strain, which was resistant to ivermectin and moxidectin, and suggested that the mutation of this gene induced resistance to macrocyclic lactones of this parasite. In addition, McCavera et al. [90] observed that the replacement of specific amino acids at codon L256, of the gene of the glutamate-gated chloride ion channels, led to decrease of ivermectin binding to the cell membrane of COS-7 cells. However, the mutation of L256 codon was not found in macrocyclic lactone-resistant *Haemonchus* strains at flock level [90]. Mutations of the genes encoding *γ*-aminobutyric acid receptor have been associated with resistance to this class of anthelmintic drugs in laboratory *H. contortus* strains [91,92,93].

Recent studies have described in detail the relationship of ivermectin with the glutamate-gated chloride ion channels in nematode helminths [94,95,96]. More precisely, the presence of glycine residue in the gene encoding these channels enhanced susceptibility of *H. contortus*, *Cooperia oncophora* and *Dirofilaria immitis*, while the presence of other residues stimulated resistance of the trematode parasites *Schistosoma mansoni*, *S. japonica* and *Clonorchis sinensis* [96]. At the same time, the mutation of this glycine residue in the glutamate-gated chloride ion a3B channels expressed on HEK293 cells, resulted in the loss of *H. contortus* ivermectin-sensitivity [94]. In contrast, Williamson et al. [64] failed to correlate macrocyclic lactone-resistance with mutations in glutamate-gated chloride ion channels and *γ*-aminobutyric acid receptors.

Several studies have highlighted the role of P-glycoprotein in the development of macrocyclic lactone-resistant *Haemonchus* strains [97]. Relevant research, after molecular analysis, has discovered polymorphisms in the P-glycoprotein genes that may be related to macrocyclic lactone resistance [98,99,100]. At the same time, the expression of P-glycoprotein appears increased in resistant parasites [64,99], while the exposure of both larvae and adult *H. contortus* in macrocyclic lactones led to overexpression of P-glycoprotein in vivo [101].

Several P-glycoprotein inhibitors can increase the sensitivity of *H. contortus* to macrocyclic lactones, both in vitro [102,103,104] and in vivo [97]. In agreement with the above findings, Lifschitz et al. [105] have concluded that P-glycoprotein inhibitors increased the efficacy of macrocyclic lactones against respectively resistant *H. contortus* strains in sheep. In general, there is evidence of multiple mechanisms for developing a resistance to avermectin [106].

### 3.4. Resistance to Closantel

Closantel is an anthelmintic drug with a limited range of action, effective against *Haemonchus*, as well as against *Fasciola*. This limited spectrum of efficacy, compared to other antiparasitic drugs, can explain the limited information about closantel-resistant parasitic strains and the relevant mechanisms of resistance. Rothwell and Sangster [107] examined closantel metabolism in closantel-susceptible and -resistant *H. contortus* and found that none of them could metabolise this drug in vitro and in vivo. However, they reported that radio-labelled closantel showed lower concentration in closantel-resistant strains compared to -susceptible ones, after administration to sheep. The same researchers concluded that closantel-resistance was attributed to the reduced closantel intake by the resistant helminths, to the strong binding of the drug to albumins in the intestine of the helminth and to the increased excretion of the drug from resistant worms [107]. Later studies have failed to detect polymorphisms in P-glycoprotein genes that could explain the reduced concentration of the drug in closantel-resistant *Haemonchus* isolates [100,108].

### 3.5. Resistance to Amino-Acetonitrile Derivatives

The possible mechanisms for developing a resistance against amino-acetonitrile derivatives (monepantel) of *Haemonchus* spp. were studied by Kaminsky et al. [109]. These researchers identified mutations in the nicotinic acetylcholine receptors genes, *Hco-des-2H* and *Hco-acr-23H* or *Hco-MPTL-1* (sites targeted by monepantel), which were associated with monepantel-resistance in *H. contortus* strains from sheep. These mutations of the monepantel receptors were detected in *H. contortus*, in experimental studies, after their exposure to high concentrations of this antiparasitic drug. More recently, field strains of monepantel-resistant *Haemonchus* have been identified by Mederos et al. [110] and Van den Brom et al. [111]. Moreover, when Bagnall et al. [112] studied mutations in the receptor protein of a highly resistant *H. contortus* population, they have concluded that some of these mutations were dominant, i.e., present in the heterozygote survivors. Another possible explanation, suggested by the same authors, was that presence of many survivors (i.e., individual helminths) resulted from the additional effect of mutations at another locus contributing to the resistance phenotype. Presence of such multiple separate mutations in the *Hco-MPTL-1* gene in the field-derived isolate may, at least partly, explain why resistance to monepantel has arisen rapidly.

### 3.6. Multi-Drug Resistance

*Haemonchus* helminths have developed resistance to all classes of anthelmintic drugs individually as detailed above, which pose challenges in the effective control of the infection. The development of multi-drug resistance in some helminths has further increased these problems.

Multi-drug resistance has been defined as the resistance to three or more different classes of anthelmintic drugs [113,114,115]. Nowadays, many publications have presented reports of identifications of strains of nematodes found to be resistant simultaneously against benzimidazoles, imidazothiazoles, macrocyclic lactones and/or monepantel. Among them, *Haemonchus* strains with multi-drug resistance have been identified in many parts of the world, Africa [116], America [117,118], Asia [119], Australia [120] and Europe [121,122].

Multi-drug resistance is the consequence of resistance development against each class of anthelmintics separately, as described above. Moreover, researchers have also described some mechanisms of potential interactions. For example, macrocyclic lactones have been found to interact with *β*-tubulin at position 200 and 167, resulting in resistant *H. contortus* against benzimidazoles [67,123]. Ivermectin, after repeated use, binds to *β*-tubulin [124], limiting the anthelmintic properties of benzimidazoles. Hence, all people involved should be aware of the potential for interactions leading to multi-drug resistance, to plan and manage relevant antiparasitic control strategies.

## 4. Challenge III: The Diagnostic Approaches

The diagnosis of haemonchosis is based on clinical signs, post-mortem findings, results of laboratory tests and outcomes of molecular methods [125].

### 4.1. Clinical Symptoms

Clinical symptoms of haemonchosis are mainly associated with anaemia, which is the result of the blood-sucking activity of the helminths [2,126]. Blood-sucking activity starts by L_4_ larvae and continues by adult helminths in the abomasum [127,128], potentially leading to clinical appearance of anaemia approximately 10 to 12 days post-infection [129]. Each adult parasite can suck about 30 to 50 μL blood per day. Therefore, a sheep infected with 1000 adult *Haemonchus* spp. would lose daily up to 50 mL of blood [130,131,132]. The intensity of the effects of haemonchosis depends on the degree of infection, the response of the immune system and the activation of hematopoiesis of the host [133]. Regarding the degree of infection and the response of the immune system of the host, haemonchosis can be divided into three forms: Hyperacute, acute and long-standing [2].

Hyperacute form of haemonchosis, although rare, is characterised by a very high burden of infection (approximately 30,000 adult parasites), which leads to severe anaemia and haemorrhagic abomasitis [134]. Deaths of the affected animals can occur suddenly, with no initial signs, while animals which survive, suffer from severe anaemia. In these animals, the predominant symptom is a distinct paleness of the mucous membranes, especially of the conjunctiva. Infected animals become gradually weak, due to the severe blood loss, and over time, they are unwilling to move. During grazing, they may collapse and die. If antiparasitic treatment is not administered, signs of hypoproteinaemia (due to severe blood loss) appear, mainly generalised subcutaneous oedema. Jaw swelling is a common clinical finding (although not unique to this infection), but still, occasionally, it may not be present. Diarrhoea is not a common sign of haemonchosis, and faeces are reduced in quantity, becoming dry and occasionally dark, due to the melaena. In cases of co-infection with other nematode parasites, diarrhoea may occur [7].

In the acute form, haemonchosis is characterised by anaemia of the infected animals, with the appearance of deaths of some of the affected animals usually four to six weeks after the initial infection. Dargie and Allonby [131] identified three stages of anaemia (I, II and III) in the acute form of haemonchosis. In stage I, mild anaemia is observed six weeks after the initial infection. Subsequently, animals that survive, present a temporary recovery, due to the activation of the haematopoietic process by the host (stage II). Finally, in stage III, a severe and persistent anaemia occurs, due to the reduction of iron stores, which further contributes to suppressing the haematopoietic process.

In the early stage of infection, haemonchosis is not directly related to reduced productivity, but if the infection is prolonged, it leads to reduced milk production, reduced average daily gain of the animals and reduced wool production. Suarez et al. [135] recorded a decrease (approximately 200 mL per sheep) in daily milk production during infections with *H. contortus* [135]. Studies have shown a 38% reduction in the mean average daily gain of lambs, nine weeks after their initial infection with *H. contortus*, followed by the onset of clinical symptoms [136]. In contrast, Van Wyk [137] did not observe a significant delay in lamb growth, despite the fact that a large number of parasitic eggs in faecal samples were found (>15,000 eggs per gram of faeces [epg]) compared to sheep in which monthly antiparasitic treatments were performed [137].

The infection of ruminants with significantly smaller numbers of *Haemonchus* spp. for a long time is characterised as long-standing haemonchosis [17,134]. This form of haemonchosis can often remain clinically unrecognised. The onset of symptoms is associated with a sudden increase of the parasitic burden or a decreased immune response, due to the poor body condition of the host. Long-standing haemonchosis occurs in areas with less favourable conditions for developing the larval stages of *Haemonchus* spp. and usually takes place with co-infection with other parasitic genera [138]. This form of *Haemonchus* spp. infection is often the result of unsuccessful anthelmintic treatments, which in combination with the development of anthelmintic resistance of *Haemonchus* spp., prevent the development of the acute form of the infection [125].

The clinical signs of long-standing haemonchosis are similar to those of malnutrition syndromes (i.e., reduction of meat and milk production), along with the appearance of anaemia in some animals. Barger and Cox [139] observed a small decrease (3%) in the average daily gain of growing sheep with good body condition score compared to sheep with poor body condition score, in which a significant reduction (>3%) of average daily gain was observed. Both groups of sheep were infected with a small number of *H. contortus* for 12 weeks [139]. Another study recorded a reduction of 30 g per day for the average daily gain in growing lambs and kids with long-standing haemonchosis [140]. Howlader et al. [141] recorded a 25% reduction in the growth rate of animals with long-standing haemonchosis. Long-standing haemonchosis led to reduction of bodyweight and wool production in Merino sheep in Australia, as well as to reduction in milk production and survival of newborn lambs [138]. Finally, long-standing haemonchosis has been found to reduce bodyweight and sudden deaths of extensively managed goats in Kenya during the period of the year with poor vegetation [142].

Special mention should be made about the FAMACHA system (Faffa Malan Chart), which is a different approach for the diagnosis of haemonchosis. Specifically, the FAMACHA system correlates the degree of conjunctival paleness with the severity of anaemia [143]. The severity of anaemia due to *Haemonchus* spp. infection is evaluated on a five-point scale, from 1 to 5 [144]. Score 1 corresponds to the normal red conjunctiva and Score 5 to a severely pale conjunctiva [144]. This system has been shown to be extremely accurate for the diagnosis of haemonchosis in areas with tropical or subtropical climatic conditions (e.g., South Africa), where small ruminant infection with *Haemonchus* dominated those by other parasitic genera [143]. Nevertheless, this system did not contribute significantly to the diagnosis of haemonchosis in areas with different climatic conditions (e.g., in areas with temperate climate), where other parasitic genera, additionally to *Haemonchus* spp., substantially infected small ruminants [145]. For example, Papadopoulos et al. [146] applied the FAMACHA system in Greece without matching the accuracy indicated by the initial authors and attributed this inaccuracy to the significant presence of *Teladorsagia circumcincta* (a nonblood-sucking parasite), which was found to be the primary nematode species in small ruminants in that country.

The clinical features of *H. contortus* infection are summarised in Table 3.

### 4.2. Post-Mortem Findings

In cases of deaths of ruminants in a farm, caused subsequently to *Haemonchus* spp. infection, post-mortem findings can allow a rapid and accurate diagnosis of the infection [125].

**Figure 1:**
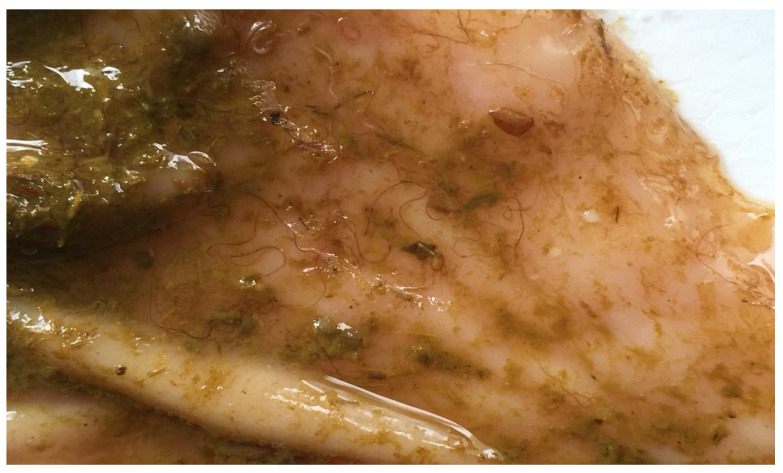
*Haemonchus* worms on the mucosa of the abomasum of an infected sheep.

The diagnosis of haemonchosis in the hyperacute form is based on two findings: The detection of large numbers of adult *Haemonchus* spp. in the abomasum (Figure 1) and the macroscopic observation of petechiae on the gastric mucosa of the organ [125].

In acute haemonchosis, cadavers of affected animals appear severely anaemic, with pale mucous membranes, ascites and submandibular oedema, indications of severe hypoproteinaemia, a result of the blood-sucking activity of *Haemonchus* spp. Blood can be unclotted and the gastric mucosa extremely oedematous, full of petechiae with visible the attached parasites [131]. In such cases, the number of helminths can vary from 2000 to 20,000 adult parasites in each animal [2].

Post-mortem findings are not indicative of long-standing haemonchosis. In such cases, for the diagnosis, the recovery of a small number of adult *Haemonchus* spp. in a dead animal’s abomasum should be coupled with a history of reduced productivity and relevant clinical signs at the population level. Nevertheless, even in such cases, clinical suspicions cannot always be confirmed by the post-mortem examination.

For the successful diagnosis of haemonchosis on post-mortem findings, knowledge of the appearance of adult *Haemonchus* would be useful. These helminths are observed on and recovered from the mucus of abomasum, which often has numerous petechiae [7]. Length is the first point for observation: The worms are large in size (2–3 cm). Further, female parasites have a characteristic turn of their white uterus around their red intestine, which can be seen filled with blood, creating a characteristic image (this has led to the parasite being named ‘barber’s pole worms’); in contrast, male worms have a completely red colour (as, obviously, the white uterus is missing). Microscopically, the male worms have long spicules (398–506 μm), while the females may have a varying vulvar flap morphology. In all individuals, there are cervical papillae on their head and a tiny lancet inside the buccal capsule, by means of which they can injure the mucosa of the abomasum. Jacquiet et al. [50] have provided detailed identification of *Haemonchus* species, particularly for sympatric helminths from the same host, based on morphometrics and characteristics of spicules.

### 4.3. Results of Laboratory Tests

**Figure 2:**
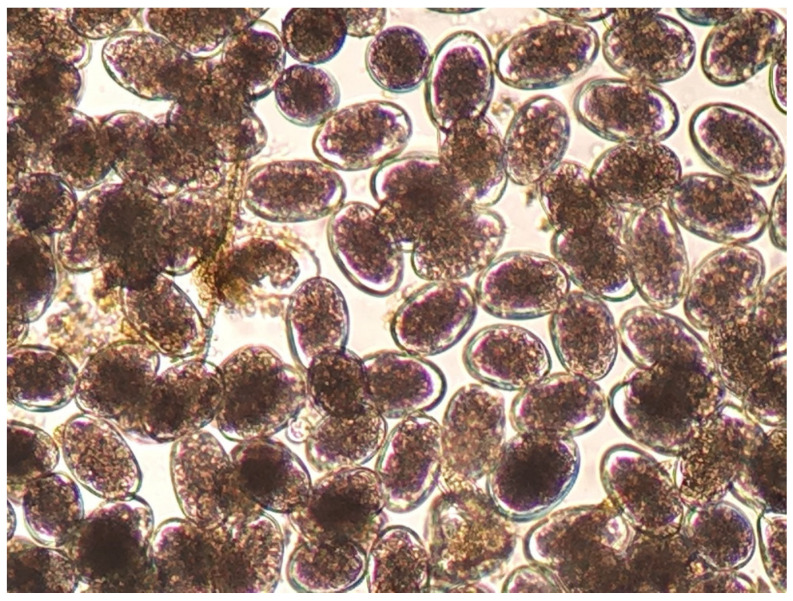
*Haemonchus* eggs as seen during microscopic observation (75 × 45 μm).

The diagnosis of haemonchosis can be confirmed by parasitological laboratory tests. For laboratory diagnosis of the infection, qualitative (e.g., Teleman method) and quantitative (e.g., modified McMaster method) parasitological tests for faecal samples can be applied, respectively, for the detection and counting of parasitic eggs [125]. Dimensions of the eggs of *Haemonchus* spp. range as follows: length 70 to 85 μm and width 41 to 48 μm; the eggs appear with 16 to 32 blastomeres. However, the morphological features of *Haemonchus* eggs (Figure 2) are not genus-specific, and hence, genus identification is difficult and practically inapplicable [7].

In experimental infections, the number of eggs excreted in the faeces has been associated with the parasitic burden of the host. According to Levine [126], 3,000 epg in faecal samples indicate a mild infection with the parasite, whilst 30,000 epg indicate a severe infection. However, under clinical conditions, single infections with this parasite are rare and, thus, this diagnostic approach has no value.

**Figure 3:**
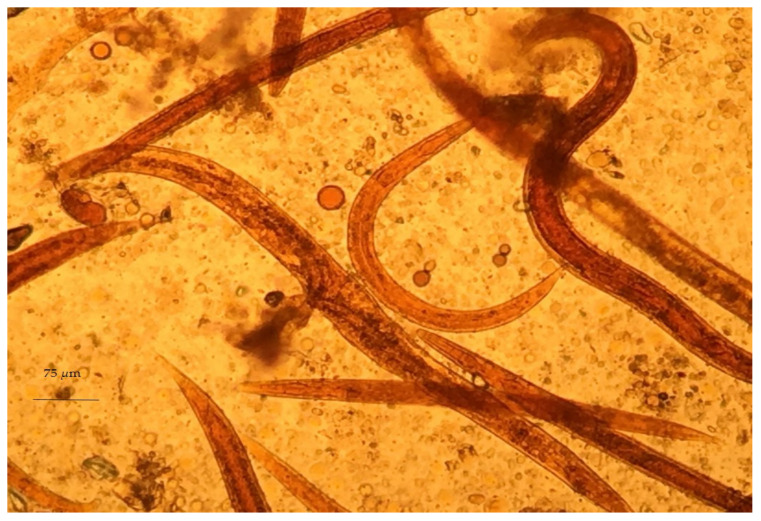
*Haemonchus* L_2_, as seen during microscopic observation.

**Figure 4:**
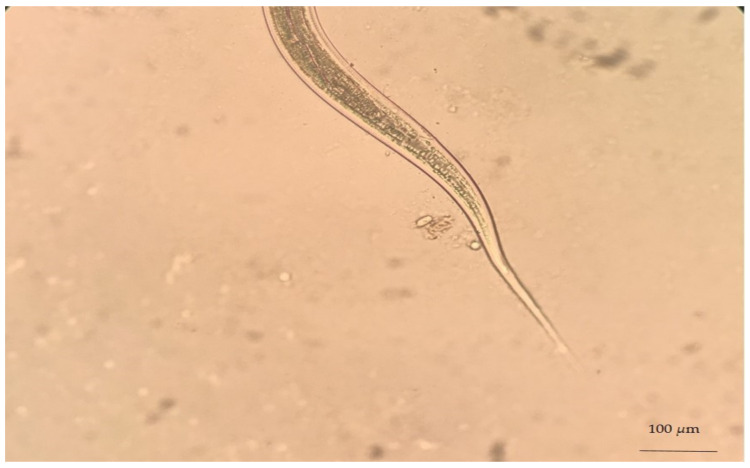
*Haemonchus* L_3_ (infective stage), as seen during microscopic observation.

Despite the difficulty of genus identification of *Haemonchus* spp. eggs, the L_3_ larvae of the parasite can easily be identified in coprocultures. A necessary precondition for the successful diagnosis of haemonchosis with this approach is the knowledge of L_3_ morphological features. First, L_3_ larvae can be distinguished by the filaroid oesophagus (L_1_ and L_2_ larvae have a bulbous oesophagus; Figure 3). Further, *Haemonchus* L_3_ larvae are recognised by the medium length of their tail (65–82 μm) (Figure 4) and the presence of 16 intestinal cells, whilst no refractile bodies are present in these [147].

A haematological examination can also assist in the diagnosis [125]. Anaemia is indicative of the infection, and acute haemonchosis has been associated with >15% decrease of the haematocrit [136] or decrease of haemoglobin concentration to less than 0.085 g mL^−1^ [148]. The haematological examination can only serve as an adjunct diagnostic technique, as on its own it is of little value to the diagnosis of the disease, given that several pathological conditions of sheep and goats can lead to reduced haematocrit and haemoglobin concentration.

### 4.4. Outcomes of Molecular Methods

Several molecular methods have been developed for the diagnosis of haemonchosis through identifying *Haemonchus* at genus and species-level [149,150,151,152,153,154]. Among these, Southern blotting [155,156] and repetitive DNA hybridisation probes in combination with Southern blots [150,151] used to be the most commonly employed ones. However, their low sensitivity and specificity contributed to their replacement by techniques based on the polymerase chain reaction (PCR).

Several genes have been targets for identification of *Haemonchus* spp. by means of conventional PCR. These have included *β*-tubulin genes, nuclear genes, as well as internal transcribed spacers (ITS), external transcribed spacers (ETS) and non-transcribed spacers (NTS) and finally mitochondrial genes, with particular emphasis on the nicotinamide dehydrogenase subunit gene 4 (*Nad4*). Roos and Grant [157] were among the first researchers, who developed a PCR for identifying *H. contortus*, targeting the isotype-1 of *β*-tubulin gene. The identification of the parasite was based on the difference in the size of the amplified gene between *H. contortus* and *Trichostrongylus colubriformis*. However, the sensitivity of the method did not prove to be high, due to PCR transfections, the large size of the target gene (1300–1500 bases) and the insufficient number of copies of the *β*-tubulin isotype 1 gene.

Several researchers have evaluated the nuclear genes of the parasite and emphasised on ITS and ETS for identifying *Haemonchus* spp. Stevenson et al. [152] were the first to evaluate ITS2 for identifying *H. contortus* and *H. placei*. After amplifying this region and its sequencing, these researchers identified several oligonucleotide polymorphisms between the two species, which contributed to their differentiation. Chaudhry et al. [154] used the above polymorphisms for the discrimination of hybrid *Haemonchus* spp., after the sequencing of ITS2. In addition, Heise et al. [158] after amplifying ITS2 with conventional PCR, sequenced it to differentiate eight different species (including *H. contortus*) of gastrointestinal nematodes. The external transcribed regions and the non-transcribed regions of nuclear DNA have also been used to identify *Haemonchus* spp. Zarlenga et al. [156] amplified these regions by conventional PCR and sequenced them. After sequencing, they detected some oligonucleotide polymorphisms in both regions, which allowed *H. contortus* to be differentiated from *H. placei*. Pichler et al. [159] have developed a snapback primer probe methodology, based on a melt curve analysis, as an effective alternative to the sequencing of the *ITS-2* gene. By use of this methodology, one can be expected to be able to identify individual adult and larval stages of *Haemonchus* spp., although this approach may not be accurate when faecal samples are used, due to the varying levels of sympatric co-infections. Finally, another gene that was used to distinguish *Haemonchus* spp. with conventional PCR is the mitochondrial *Nad4*. Blouin et al. [160] amplified this *Nad4* in adult *H. contortus* and *H. placei* and sequenced them and identified differences in the sequences between these two species, concluding that the mitochondrial *Nad4* could be used for identification of *H. contortus* and *H. placei*, and thus, the diagnosis of the respective infections [160,161].

Nevertheless, the conventional PCR cannot overcome the following two limitations for identifying nematode parasites at the species level in faecal samples, by means of targeting the extracted DNA of parasitic eggs: (a) presence of ‘pure’ DNA with no PCR inhibitors and (b) sufficient amount of DNA of the target parasite to achieve detection. As a result, it is characterised by low sensitivity, attributed to the combined presence of various PCR inhibitors in faecal samples and the small amount of extracted DNA of the parasite therein [162,163].

Real-time PCR aims to quantify the transcription of the target gene. As a result, it was applied for identification of *Haemonchus* spp. and also for measurement of the parasitic burden [164,165,166,167,168]. Siedek et al. [165], using real-time PCR, were able to identify L_3_ of *H. contortus* in coprocultures, targeting on ITS2. Several researchers have used this method to identify gastrointestinal nematode eggs in faecal samples, by means of amplifying ITS2 and 28S genes of ribosomal DNA [169,170,171]. Similarly, Sweeny et al. [172] were able to identify three different species (including *H. contortus*) and two different genera of nematodes, targeting on ITS2 from DNA extracted from parasitic eggs. However, these researchers recorded increased sensitivity and specificity of the technique with >50 epg in faecal samples. Later, McNally et al. [168] developed a real-time PCR based on ITS2, which could differentiate parasitic eggs of *Haemonchus*, *Trichostrongylus* and *Teladorsagia* in faecal samples from sheep, as well as count them. This technique was reported to have a sensitivity of 10 epg. However, given the abundance of PCR inhibitors in the samples, the researchers proposed its application after a cleaning process of the parasitic eggs therein [163,173].

To overcome the possible limitations of the above PCRs, Elmahalawy et al. [174] have developed a Droplet Digital PCR (ddPCR). Their results clearly indicated that ddPCR was a suitable choice for detection and absolute quantification of the major genera of helminths, which parasitised sheep; they also found that the technique could be used in samples with helminths of many species and in larval cultures from pooled faecal samples from sheep. These findings confirm that ddPCR can overcome limitations of the conventional PCR and can be a useful complement to applications based on conventional egg counting methods.

## 5. Challenge IV: Future Control

### 5.1. Pharmaceutical Control

There are several classes of anthelmintic drugs effective against *Haemonchus*; these include benzimidazoles (e.g., albendazole), imidazothiazoles (e.g., levamisole), macrocyclic lactones (e.g., ivermectin), salicylanides (e.g., closantel), amino-acetonitrile derivatives (e.g., monepantel), sporoindoles (e.g., derquantel) [175,176]. Commercially available preparations may include the above as single active ingredients or in combinations.

In recent years, due to the widespread development of anthelmintic resistance of *Haemonchus* spp., special emphasis has been given to control and prevention of haemonchosis with the application of both targeted treatments [177,178] and targeted selective treatments [179]. These approaches aim to keep the largest possible numbers of *Haemonchus* spp. *in refugia*, thus maintaining the susceptibility of these parasites to anthelmintic drugs [179]. *In refugia* is defined as the part of the parasitic population that is not exposed to anthelmintic drugs, i.e., individuals at a free-living (non-parasitic) stage of their biological cycle or individuals that live within a host but still not exposed to anthelmintic treatment [179,180].

More precisely, targeted treatment is the administration of anthelmintic treatment to all sheep or goats in a farm at specific times-points, to keep as high parasitic population as possible *in refugia* [181]. This approach intends to reduce the number of anthelmintic treatments in a flock and, therefore, to minimise the development of resistance in parasites at pasturelands. Proper application of targeted treatments results in the increased interval between two anthelmintic administrations. Thus, *Haemonchus* spp. populations susceptible to anthelmintic drugs have more time available to infect a host and start laying eggs, thus, to recover in numbers and re-infect pasturelands with susceptible strains; for example, targeted treatments in Australia [177] and Italy [178] are discontinued during the summer months, because the parasitic burden in the pasturelands (*in refugia*) would be reduced during these periods.

The distribution of parasites among animals in a sheep/goat farm is not homogeneous. The majority of the helminths are infecting only a few animals [182,183]; for example, Sréter et al. [184] concluded that 80% of the parasites were detected in 20% to 30% of the animals, while the majority of the animals were infected with only low parasitic burdens. Animals with increased parasitic burdens would be responsible for the spread of nematode larvae in natural pasturelands, as well as usually also showing clinical disease. In addition, the presence of parasitic burden among animals in a farm has a repeatability pattern, i.e., the same animals in the farm seem to develop severe parasitic infections, requiring repeated anthelmintic treatments [143,182]. The principle of targeted selective treatments is based on the administration of anthelmintics exclusively to animals that need them and would benefit from them, in terms of health and productivity, leaving the rest of the animals in the same flock untreated [179]. The expected advantages of this approach would be the delay of development of anthelmintic resistance, due to the lower exposure of parasites to anthelmintic drugs, the reduced residues of anthelmintic drugs in meat and milk of animals and the smaller costs of treatments [185]. On the other hand, one significant disadvantage of targeted selective treatments is the possibility that some highly infected animals may escape the anthelmintic treatment, resulting to developing clinical diseases and production losses, as well as disseminating the infection.

Various criteria have been proposed to select animals for anthelmintic administration, as part of targeted selective treatment. These include parasitological (e.g., increased epg in faecal samples), clinical (e.g., presence of anaemia detected by the FAMACHA system [144], presence of diarrhoea [186] or low body condition score [187]) or production (e.g., reduced milk production [188]) criteria. After animals for administration of anthelmintics would be selected based on the above criteria, then one could also select animals to remain untreated, taking into account that at least 10% of the infected animals in a farm should remain untreated, in order to maintain *Haemonchus* spp. *in refugia*. Leaving a proportion of sheep in a flock untreated was found to be effective in delaying the development of anthelmintic resistance; a proportion as low as 10% of a flock untreated was sufficient to significantly delay developing a anthelmintic resistance [189,190]. These animals should be allocated within each grazing group, not just across the flock.

### 5.2. Non-Chemical Means of Control

Protocols for control of haemonchosis by non-pharmaceutical means include (a) grazing management, emphasising on rotational grazing [191,192,193,194], (b) modification of animals’ diet [195], which directly affects the response of the animals’ immune system to haemonchosis [196], (c) selection of animals genetically-resistant against *Haemonchus* spp. [197,198] and (d) administration of various products to animals, e.g., nematophagous fungi [199,200], tannins [201,202] or polyphenolic compounds [201,203,204].

Grazing management, cell or rotational grazing, contributes to minimising the intake of the infective larvae of *Haemonchus* spp. by susceptible sheep and also limiting excessive contamination of pasturelands with parasitic eggs, that way decreasing the risk of infection. Studies on this approach confirmed the beneficial effect of rotational grazing against haemonchosis, despite the necessity for differing schedules in accord to the various environments [35,205,206]. A possible failure to controlling the infection through grazing management can be due to a possible lack of awareness for its potential, as well as for practical reasons, as animals’ movements are determined by nutritional availability [207].

The ability of ruminants to resist parasitic infections when they are kept in good nutritional status (nutritional management) has been confirmed [208,209]. It has been established that sheep given a low protein diet seem to be more susceptible to *Haemonchus* spp. infections compared to animals given supplementary protein [210,211,212]. Therefore, there is a relation between nutritional management and resistance to haemonchosis, which can be beneficial when it is planned while considering the epidemiology of the helmiths [213] and is applied on its own or in combination with other control strategies [214].

Many plants contain bioactive compounds, which have been associated with reduced parasitic burdens and increased animal production. Among them, polyphenolic compounds, tannins, in particular, are characterised with antiparasitic properties. These can act directly against the helminths at various stages of their life-cycles [215], or they improve the immune response of the animals, reducing the shedding rate [216]. However, there are limitations in their use, as they can lead to detrimental nutritional effects, if fed in excessive rates or when protein intake is low [217].

The use of nematophagous fungi was a promising alternative for use against resistant *Haemonchus* strains. These have the ability to trap and destroy infective larvae of the helminth, after the formation of hyphae. Specifically, the fungal spores fed to the animals, develop during their passage through the gastrointestinal tract of sheep and predate the infective larvae [218]. Among the most widely evaluated fungal species was *Duddingtonia flagrans* [219], trials with which showed results supporting their use in sheep or goats for control of *H. contortus* [220,221,222].

Sheep of some breeds (e.g., Morada Nova, Red Maasai, Barbados Black Belly) resistant (or even resilient) to haemonchosis can be used as an interesting option to minimise adverse effects of *Haemonchus* infection. Further, as such animals harbour fewer worms than ones of susceptible breeds, they also excrete a smaller number of eggs with faeces and do not contribute to intense infection of pastures. Finally, these animals can contribute to controlling anthelmintic resistance, as they would require less frequent anthelmintic treatments [223,224,225,226,227]. The introgression of these resistant breeds, through crossbreeding, could serve as a desirable alternative, but should be done with prudence, to maintain the production traits of the existing flock.

In the same direction, the possible selection of individuals, within a certain breed, resistant (or resilient) against *Haemonchus* spp. can also be an option [228,229]. These genetic selection strategies can be facilitated by using traits with relatively high heritability (e.g., faecal egg counts [FEC], haematocrit, body condition). For example, immunity against *H. contortus* expressed as FEC and packed cell volume values is a heritable trait in sheep [230], and there are differences among different breeds in *Haemonchus* infection. However, some studies in Merino sheep breeds have concluded that selection for low *H. contortus* FEC may also lead to lower animal production [228,231]. The possibility for more accurate and easily used genetic markers has been widely investigated, including quantitative trait loci, algorithms based on haematological parameters, immunological indicators and molecular markers. Currently, there is one genomic test commercially available (Wormstar, Zoetis), by means of which the resistance variation among individual sheep can be assessed. Potentially, in the future, a potential technology could is the development and commercial availability of sufficient numbers of transgenic animals able to resist the infection [232].

All the above approaches, however, have produced mixed results and, in general, for none of them, results matching the efficacy of anthelmintic drugs have been reported. Hence, they would be used mainly as support options.

### 5.3. Vaccination

Only recently, vaccines have become available against *Haemonchus* infection [233,234]. An effective vaccine against *H. contortus* has been produced for use in sheep, using the latent H11 antigen [235], which is a protein of the gastrointestinal tract of *H. contortus*, and H-gal-GP, which were extracted from adult *H. contortus*. Clinical trials have confirmed the efficacy of the vaccine (Barbervax^®^), which was developed at the Moredun Research Institute in Edinburgh; for example, significant protection against *H. contortus* in lambs was indicated after repeated administration with three-week intervals, although findings did not confirm full efficacy in ewes after severe experimental infection [236]. The vaccine is now produced in Australia [237], where it has been licenced for commercial use in late 2014.

## 6. Concluding Remarks: Should We Worry?

*H. contortus* is a highly pathogenic nematode parasite of ruminants which, due to its blood-sucking activity, results in severe problems in sheep and goat farms. Climatic conditions play an important role and have a significant impact on the development of this infection. In areas with humid and warm climatic conditions, infection with *Haemonchus* spp. is enabled and facilitated. Within this context, recent epidemiological studies have suggested an alarming extension to the geographical distribution of significant *H. contortus* infection, particularly in colder temperate climates in the Northern Hemisphere. Coupling this with the existing resistance of *Haemonchus* strains to the various anthelmintics of all classes is a cause of worry, as it should be taken constantly into account in the formulation of health plans for control of the infection. One should add to that the difficulties in the diagnosis of the disease: Field evidence provides suspicion about infection, which needs to be subsequently confirmed by laboratory tests through parasitological or molecular techniques.

Considering the above, control of the infection should be based in the correct application of plans that include targeted treatments and selectively targeted treatments of administration of anthelmintic drugs, given that the seemingly effective vaccine does not hold a wide licence and can only protect against one parasitic species (meaning that administration of pharmaceuticals would need to continue to control other parasitic pathogens). A transdisciplinary approach bringing together veterinary parasitologists, with scientists from other fields, e.g., livestock scientists, grassland-management experts, epidemiologists and clinicians, would be needed to be adopted for better and sustainable parasite control [238].

To answer the main question of this study: ‘Should we worry?’ ‘*No, but we should be concerned*’.

## Figures and Tables

**Table 1:** Summary of epidemiological features of *Haemonchus contortus* infections according to climatic regions.

Climatic Zones	Regions	Ecological Features	Parasite Epidemiology
Tropical and subtropical regions	Tropical regions of Africa and America, tropical islands of the Pacific Ocean, southern and south-east Asia, northern part of Australia, southern USA, the Caribbean	L_3_ not surviving on pasturelands for long; moisture allowing larval development during dry seasons; increased period of larval survival and development when adequate moisture	Larval populations developing constantly, and animals continuously challenged; in dry weather, L_3_ increasing seasonally; hypobiotic L_4_ developing during dry seasons
Warm temperate and summer rainfall regions	Parts of southern USA and South America, southern and eastern Asia, southern Africa, eastern Australia	Combination of high temperature/moisture supporting development of L_3_; under cold conditions, larval survival and development slowing down	Significant problem, depending on rainfalls; when mild temperatures during winter, L_3_ potentially throughout a year; in areas with low temperatures, outbreaks depending on seasonality; hypobiosis predominating during cold winters
Mediterranean climatic regions	South-west cape of South Africa, para-Mediterranean basin, south-east Australia, western Australia	Suspension of survival and development of free-living stages; larval populations reaching peak numbers in autumn and spring; during mild temperature in winter, possible survival of L_3_	Highest populations from late autumn to early winter and late spring to early summer; varying hypobiosis, in accord with duration and intensity of hot/dry conditions
Cool and cold temperate regions	Northern USA and Canada, New Zealand, south east-Australia, northern Europe	Cessation of larval development until the onset of milder environmental conditions	Low risk, limited to warmer months; hypobiosis permitting overwintering; high temperatures favouring rapid development of hypobiotic larvae
Arid regions	Deserts of southern and sub-Saharan Africa, continental Australia, Middle East	Lack of moisture limiting survival and development of larval populations, favoured during rainfall periods	Not a significant threat; rainfall periods increasing larval availability; hypobiosis of varying importance; hot conditions reducing L_3_

**Table 2:** Summary of mechanisms for the resistance of *H. contortus* against the various anthelmintic drugs.

Class of Anthelmintic Drugs	Target in Helminth	Mechanism
Benzimidazoles	*β*-tubulin (isotype-1)	Point mutation at codon 200 leading to a phenylalanine to tyrosine substitution
		Point mutation at codon 167 leading to a phenylalanine to tyrosine substitution
		Point mutation at codon 198 leading to an alanine to glutamic acid substitution
	*β*-tubulin (isotype-2)	Attribution to polymorphism(s)
Imidazothiazoles	Nicotinic acetylcholine receptor genes	Mutated genes (*Hco-unc-63, Hco-acr-8*) expressing production of a protein, binding to the nicotinic acetyl-choline receptors, preventing binding of drug on them
		Under-expression of certain genes (*Hco-unc-63a, Hco-unc-29.3,* etc.) encoding the nicotinic acetylcholine receptors
Macrocyclic lactones	Glutamate-gated chloride ion channels	Presence of glycine residue in the gene encoding these channels enhancing susceptibility of *H. contortus*; mutation of this glycine residue on HEK293 cells, resulting in loss of *H. contortus* drug-sensitivity
	P-glycoprotein gene	Polymorphisms or over-expression of P-glycoprotein genes
Closantel		Resistance attributed to reduced closantel intake by resistant helminths, to strong binding of the drug to albumins in the intestine of helminths and to increased excretion of the drug from resistant helminths
Monepantel	Nicotinic acetylcholine receptor genes	Mutated genes (*Hco-des-2H*, *Hco-acr-23H*, *Hco-MPTL-1*) associated with resistance

**Table 3:** Summary of the clinical features of *H. contortus* infection.

	Features
Hyperacute	Severe anaemia, sudden deaths, paleness of mucous membranes, weakness, un-willingness to move, subcutaneous oedemas, dry, dark faeces of reduced quantity
Acute	Stage I: Death of affected animals, mild anaemia in surviving animals
	Stage II: Temporary recovery due to activation of the haematopoietic process by host
	Stage III: Severe and persistent anaemia due to iron deficiency
Subacute	Decreased growth rate, reduced milk, meat, wool production
Long-standing	Features of malnutrition, e.g., low body condition score, decreased growth rate, reduced milk, meat, wool production

## Data Availability

Review article with no new data included therein.

## Appendix A

*Haemonchus* species identified and described, with ruminant species from which they have been respectively recovered (Hoberg et al. [239]).

***Haemonchus* Species**	**Ruminant Species from Which** **They Have Been Recovered**	**Geographical Distribution**
*H. bedfordi*	african buffaloes, giraffes, goats, sheep	Mainly Africa
*H. contortus*	antelopes, bovines, cervidae, giraffes, goats, sheep, wild ruminants	Universal
*H. dinniki*	wild ruminants	Mainly Africa
*H. horaki*	wild ruminants	Mainly Africa
*H. krugeri*	wild ruminants	Mainly Africa
*H. lawrencei*	wild ruminants	Mainly Africa
*H. longistipes*	antelopes, cattle, camels	Mainly Africa, Europe, Asia
*H. mitchelli*	goats, wild ruminants	Mainly Africa
*H. okapiae*	wild ruminants	Mainly Africa
*H. placei*	antelopes, buffaloes, cattle, cervidae, sheep, wild ruminants	Universal
*H. similis*	bisons, buffaloes, cattle, cervidae, sheep, wild ruminants	Universal
*H. vegliai*	goats, wild ruminants	Mainly Africa

## References

[1] Soulsby E.J.L. (1968). Helminths, Arthropods and Protozoa of Domestic Animals.

[2] Urquhart G.M., Armour J., Duncan J.L., Dunn A.M., Jennings F.W. (1996). Veterinary Parasitology.

[3] Brasil B.S.A.F., Ronaldo L., Nunes R.L., Bastianetto E., Drummond M.G., Carvalho D.C., Leite R.C., Molento M.B., Oliveira D.A.A. (2012). Genetic diversity patterns of *Haemonchus placei* and *Haemonchus contortus* populations isolated from domestic ruminants in Brazil. Int. J. Parasitol..

[4] Hussain T., Periasamy K., Nadeem A., Ellahi M.B., Pichler R., Diallo A. (2014). Sympatric species distribution, genetic diversity and population structure of *Haemonchus* isolates from domestic ruminants in Pakistan. Vet. Parasitol..

[5] Akkari H., Jebali J., Gharbi M., Mhadhbi M., Awadi S., Darghouth M.A. (2013). Epidemiological study of sympatric *Haemonchus* species and genetic characterization of *Haemonchus contortus* in domestic ruminants in Tunisia. Vet. Parasitol..

[6] Dos Santos M.C., Amarante M.R.V., Amarante A.F.T. (2020). Is there competition between *Haemonchus contortus* and *Haemonchus placei* in a pasture grazed by only sheep?. Vet. Parasitol..

[7] Taylor M.A., Coop R.L., Wall R.L. (2007). Veterinary Parasitology.

[8] Fetterer R.H., Rhoads M.L. (1998). A hemolytic factor from *Haemonchus contortus* alters erythrocyte morphology. Vet. Parasitol..

[9] Fthenakis G.C., Papadopoulos E. (2018). Impact of parasitism in goat productions. Small Rumin. Res..

[10] Miller J.E., Kaplan R.M., Pugh D.G., Pugh D.G., Baird A.N. (2011). Internal parasites. Sheep and Goat Medicine.

[11] McRae K., McEwan J.C., Dodds K.G., Gemmell N.J. (2014). Signatures of selection in sheep bred for resistance or susceptibility to gastrointestinal nematodes. Genomics.

[12] Gordon H.M. (1948). The epidemiology of parasitic diseases with special reference to studies with nematode parasites of sheep. Aust. Vet. J..

[13] Rossanigo C.E., Gruner L. (1995). Moisture and temperature requirements in faeces for the development of free-living stages of gastrointestinal nematodes of sheep, cattle and deer. J. Helminthol..

[14] Silverman P.H., Campell J.A. (1959). Studies on parasitic worms of sheep in Scotland. I. Embryonic and larval development of *Haemonchus contortus* at constant conditions. Parasitology.

[15] Eysker M., Kooyman F.N. (1993). Notes on necropsy and herbage processing techniques for gastrointestinal nematodes of ruminants. Vet. Parasitol..

[16] Waller P.J., Thomas R.J. (1975). Field studies on inhibition of *Haemonchus contortus*. Parasitology.

[17] Allonby E.W., Urquhart G.M. (1975). The epidemiology and pathogenic significance of haemonchosis in a Merino flock in East Africa. Vet. Parasitol..

[18] Barger I.A. (1999). The role of epidemiological knowledge and grazing management for helminth control in small ruminants. Int. J. Parasitol..

[19] Dorny P., Symoens C., Jalila A., Vercruysse J., Sani R. (1995). Strongyle infections in sheep and goats under the traditional husbandry system in peninsular Malaysia. Vet. Parasitol..

[20] Cheah T.S., Rajamanickam C. (1997). Epidemiology of gastro-intestinal nematodes of sheep in wet tropical conditions in Malaysia. Trop. Anim. Health Prod..

[21] Chandrawathani P. (2004). Problems in the Control of Nematode Parasites of Small Ruminants on Malaysia: Resistance to Anthelmintics and the Biological Control Alternatives. Ph.D. Thesis.

[22] Vercruysse J. (1983). A survey of seasonal changes in nematode faecal egg count levels of sheep and goats in Senegal. Vet. Parasitol..

[23] Fritsche T., Kaufman J., Pfister K. (1993). Parasite spectrum and seasonal epidemiology of gastrointestinal nematodes of small ruminants in the Gambia. Vet. Parasitol..

[24] Ndamukong K.J.N., Ngone M.M. (1996). Development and survival of *Haemonchus contortus* and *Trichostrongylus* sp. on pasture in Cameroon. Trop. Anim. Health Prod..

[25] Nginyi J.M., Duncan J.L., Mellor D.J., Stear M.J., Wanyangu S.W., Bain R.K., Gatongi P.M. (2001). Epidemiology of parasitic gastrointestinal nematode infections of ruminants on smallholder farms in central Kenya. Res. Vet. Sci..

[26] Nwosu C.O., Madu P.P., Richards W.S. (2007). Prevalence and seasonal changes in the population of gastrointestinal nematodes of small ruminants in the semi-arid zone of north-eastern Nigeria. Vet. Parasitol..

[27] Sissay M.M., Uggla A., Waller P.J. (2007). Epidemiology and seasonal dynamics of gastrointestinal nematode infections of sheep in a semi-arid region of eastern Ethiopia. Vet. Parasitol..

[28] Bolajoko M.B., Morgan E.R. (2012). Relevance of improved epidemiological knowledge to sustainable control of *Haemonchus contortus* in Nigeria. Anim. Health Res. Rev..

[29] Blackie S. (2014). A review of the epidemiology of gastrointestinal nematode infections in sheep and goats in Ghana. J. Agric. Sci..

[30] Vercruysse J. (1985). The seasonal prevalence of inhibited development of *Haemonchus contortus* in sheep in *Senegal*. Vet. Parasitol..

[31] Okon E.D., Enyenihi U.K. (1977). Development and survival of *Haemonchus contortus* on pastures in Ibadan. Trop. Anim. Health Prod..

[32] Pandey V.S. (1990). *Haemonchus contortus* with low inhibited development in sheep from the Highveld of Zimbabwe. Vet. Parasitol..

[33] Miller J.E., Bahirathan M., Lemarie S.L., Hembry F.G., Kearney M.T., Barras S.R. (1998). Epidemiology of gastrointestinal nematode parasitism in Suffolk and Gulf Coast Native sheep with special emphasis on relative susceptibility to *Haemonchus contortus* infection. Vet. Parasitol..

[34] Southcott W.H., Major G.W., Barger I.A. (1976). Seasonal pasture contamination and availability of nematodes for grazing sheep. Aust. J. Agric. Res..

[35] Bailey J.N., Kahn L.P., Walkden-Brown S.W. (2009). Availability of gastro-intestinal nematode larvae to sheep following winter contamination of pasture with six nematode species on the Northern Tablelands of New South Wales. Vet. Parasitol..

[36] Swan R.A. (1970). The epizootiology of haemonchosis in sheep. Aust. Vet. J..

[37] De Chaneet G.C., Mayberry C.J. (1978). Ovine Haemonchosis: A Review and Report of Epizootics in North-West Western Australia and of a Trial at Esperance Western Australia.

[38] Rossiter L.W. (1964). The epizootiology of nematode parasites of sheep in the coastal area of the Eastern Province. Onderstepoort J. Vet. Res..

[39] Muller G.L. (1968). The epizootiology of helminth infestations of sheep in the South-Western districts of the Cape. Onderstepoort J. Vet. Res..

[40] Uriarte J., Valderrabano J. (1989). An epidemiological study of parasitic gastroenteritis in sheep under an intensive grazing system. Vet. Parasitol..

[41] Brunsdon R.V. (1970). Seasonal changes in the level and composition of nematode worm burdens in young sheep. N. Z. Vet. J..

[42] Vlassoff A. (1973). Seasonal availability of infective trichostrongyle larvae on pasture grazed by lambs. N. Z. J. Exp. Agric..

[43] Brunsdon R.V. (1973). Inhibited development of *Haemonchus contortus* in naturally acquired infections in sheep. N. Z. Vet. J..

[44] McKenna P.B. (1974). The persistence and fate of inhibited *Haemonchus contortus* larvae in young sheep. N. Z. Vet. J..

[45] Waller P.J., Rudby-Martin L., Ljungstrom B.L., Rydzik A. (2004). The epidemiology of abomasal nematodes of sheep in Sweden, with particular reference to over-winter survival strategies. Vet. Parasitol..

[46] Sargison N.D., Wilson D.J., Bartley D.J., Penny C.D., Jackson F. (2007). Haemonchosis and teladorsagiosis in a Scottish sheep flock putatively associated with the overwintering of hypobiotic fourth stage larvae. Vet. Parasitol..

[47] Viljoen J.H. (1969). Further studies on the epizootiology of nematode parasites of sheep in the Karoo. Onderstepoort J. Vet. Res..

[48] Biggs H.C., Anthonissen M. (1982). The seasonal incidences of helminth parasites and *Oestrus ovis* in Karakul sheep in the Kalahari region of South West Africa-Namibia. Onderstepoort. J. Vet. Res..

[49] El-Azazy O.M.E. (1995). Seasonal changes and inhibited development of the abomasal nematodes of sheep and goats in Saudi Arabia. Vet. Parasitol..

[50] Jacquiet P., Humbert J.F., Comes A.M., Cabaret J., Thiam A., Cheikh D. (1995). Ecological, morphological and genetic characterization of sympatric *Haemonchus* spp. parasites of domestic ruminants in Mauritania. Parasitology.

[51] Altaif K.I., Yakoob A.Y. (1987). Development and survival of *Haemonchus contortus* larvae on pasture in Iraq. Trop. Anim. Health Prod..

[52] Eysker M., Bakker N., Kooyman F.N.J., Van der Linden D., Schrama C., Ploeger H.W. (2005). Consequences of the unusually warm and dry summer of 2003 in The Netherlands: Poor development of free living stages, normal survival of infective larvae and long survival of adult gastrointestinal nematodes of sheep. Vet. Parasitol..

[53] Kenyon F., Sargison N.D., Skuce P., Jackson F. (2009). Sheep helminth parasitic disease in south eastern Scotland arising as a possible consequence of climate change. Vet. Parasitol..

[54] Polley L., Hoberg E., Kutz S. (2010). Climate change, parasites and shifting boundaries. Acta Vet. Scand..

[55] Kotze A.C., Prichard R.K. (2016). Anthelmintic resistance in *Haemonchus contortus*: History, mechanisms and diagnosis. Adv. Parasitol..

[56] Lacey E., Prichard R.K. (1986). Interactions of benzimidazoles (BZ) with tubulin from BZ-sensitive and BZ-resistant isolates of *Haemonchus contortus*. Mol. Biochem. Parasitol..

[57] Lubega G.W., Prichard R.K. (1990). Specific interaction of benzimidazole anthelmintics with tubulin: High-affinity binding and benzimidazole resistance in *Haemonchus contortus*. Mol. Biochem. Parasitol..

[58] Kwa M.S.G., Veenstra J.G., Roos M.H. (1994). Benzimidazole resistance in *Haemonchus contortus* is correlated with a conserved mutation at amino acid 200 in [beta]-tubulin isotype-1. Mol. Biochem. Parasitol..

[59] Kwa M.S., Veenstra J.G., Van Dijk M., Roos M.H. (1995). Beta-tubulin genes from the parasitic nematode *Haemonchus contortus* modulate drug resistance in *Caenorhabditis elegans*. J. Mol. Biol..

[60] Elard L., Comes A.M., Humbert J.F. (1996). Sequences of beta-tubulin cDNA from benzimidazole-susceptible and -resistant strains of *Teladorsagia circumcincta*, a nematode parasite of small ruminants. Mol. Biochem. Parasitol..

[61] Elard L., Cabaret J., Humbert J.F. (1999). PCR diagnosis of benzimidazole-susceptibility or -resistance in natural populations of the small ruminant parasite, *Teladorsagia circumcincta*. Vet. Parasitol..

[62] Prichard R. (2001). Genetic variability following selection of *Haemonchus contortus* with anthelmintics. Trends Parasitol..

[63] Pape M., Posedi J., Failing K., Schnieder T., von Samson-Himmelstjerna G. (2003). Analysis of the beta-tubulin codon 200 genotype distribution in a benzimidazole susceptible and resistant cyathostome population. Parasitology.

[64] Williamson S.M., Storey B., Howell S., Harper K.M., Kaplan R.M., Wolstenholme A.J. (2011). Candidate anthelmintic resistance-associated gene expression and sequence polymorphisms in a triple-resistant field isolate of *Haemonchus contortus*. Mol. Biochem. Parasitol..

[65] Ghisi M., Kaminsky R., Maser P. (2007). Phenotyping and genotyping of *Haemonchus contortus* isolates reveals a new putative candidate mutation for benzimidazole resistance in nematodes. Vet. Parasitol..

[66] Kotze A.C., Cowling K., Bagnall N.H., Hines B.M., Ruffell A.P., Hunt P.W., Coleman G.T. (2012). Relative level of thiabendazole resistance associated with the E198A and F200Y SNPs in larvae of a multi-drug resistant isolate of *Haemonchus contortus*. Int. J. Parasitol. Drugs Drug Resist..

[67] Mottier M.L., Prichard R.K. (2008). Genetic analysis of a relationship between macrocyclic lactone and benzimidazole anthelmintic selection on *Haemonchus contortus*. Pharmacogenet. Genom..

[68] Barrere V., Alvarez L., Suarez G., Ceballos L., Moreno L., Lanusse C., Prichard R.K. (2012). Relationship between increased albendazole systemic exposure and changes in single nucleotide polymorphisms on the beta-tubulin isotype 1 encoding gene in *Haemonchus contortus*. Vet. Parasitol..

[69] Silvestre A., Sauve C., Corter J., Cabaret J. (2009). Contrasting genetic structures of two parasitic nematodes, determined on the basis of neutral microsatellite markers and selected anthelmintic resistance markers. Mol. Ecol..

[70] Geary T.G., Nulf S.C., Favreau M.A., Tang L., Prichard R.K., Hatzenbuhler N.T., Shea M.H., Alexander S.J., Klein R.D. (1992). Three beta-tubulin cDNAs from the parasitic nematode *Haemonchus contortus*. Mol. Biochem. Parasitol..

[71] Beech R.N., Prichard R.K., Scott M.E. (1994). Genetic variability of the beta-tubulin genes in benzimidazole-susceptible and -resistant strains of *Haemonchus contortus*. Genetetics.

[72] Lubega G.W., Klein R.D., Geary T.G., Prichard R.K. (1994). *Haemonchus contortus*: The role of two beta-tubulin gene subfamilies in the resistance to benzimidazole anthelmintics. Biochem. Pharmacol..

[73] Saunders G.I., Wasmuth J.D., Beech R., Laing R., Hunt M., Naghra H., Cotton J.A., Berriman M., Britton C., Gilleard J.S. (2013). Characterization and comparative analysis of the complete *Haemonchus contortus* b-tubulin gene family and implications for benzimidazole resistance in strongylid nematodes. Int. J. Parasitol..

[74] Rufener L., Kaminsky R., Mäser P. (2009). In vitro selection of *Haemonchus contortus* for benzimidazole resistance reveals a mutation at amino acid 198 of beta-tubulin. Mol. Biochem. Parasitol..

[75] Sangster N.C., Davis C.W., Collins G.H. (1991). Effects of cholinergic drugs on longitudinal contraction in levamisole-susceptible and -resistant *Haemonchus contortus*. Int. J. Parasitol..

[76] Sangster N.C., Riley F.L., Wiley L.J. (1998). Binding of [3H]m-aminolevamisole to receptors in l evamisole-susceptible and -resistant *Haemonchus contortus*. Int. J. Parasitol..

[77] Sangster N.C., Gill J. (1999). Pharmacology of anthelmintic resistance. Parasitol. Today.

[78] Sangster N.C., Redwin J.M., Bjorn H. (1998). Inheritance of levamisole and benzimidazole resistance in an isolate of *Haemonchus contortus*. Int. J. Parasitol..

[79] Neveu C., Charvet C., Fauvin A., Cortet J., Castagnone-Sereno P., Cabaret J. (2007). Identification of levamisole resistance markers in the parasitic nematode *Haemonchus contortus* using a cDNA-AFLP approach. Parasitology.

[80] Fauvin A., Charvet C., Issouf M., Cortet J., Cabaret J., Neveu C. (2010). cDNA-AFLP analysis in levamisole-resistant *Haemonchus contortus* reveals alternative splicing in a nicotinic acetylcholine receptor subunit. Mol. Biochem. Parasitol..

[81] Neveu C., Charvet C., Fauvin A., Cortet J., Beech R., Cabaret J. (2010). Genetic diversity of levamisole receptor subunits in parasitic nematode species and abbreviated transcripts associated with resistance. Pharmacogenet. Genom..

[82] Boulin T., Fauvin A., Charvet C., Cortet J., Cabaret J., Bessereau J.-L., Neveu C. (2011). Functional reconstitution of *Haemonchus contortus* acetylcholine receptors in *Xenopus* oocytes provides mechanistic insights into levamisole resistance. Br. J. Pharmacol..

[83] Barrere V., Beech R.N., Charvet C.L., Prichard R.K. (2014). Novel assay for the detection and monitoring of levamisole resistance in *Haemonchus contortus*. Int. J. Parasitol..

[84] Dos Santos J.M.L., Vasconcelos J.F., Frota G.A., de Freitas E.P., Teixeira M., da Silva Vieira L., Bevilaqua C.M.L., Monteiro J.P. (2019). Quantitative molecular diagnosis of levamisole resistance in populations of *Haemonchus contortus*. Exp. Parasitol..

[85] Sarai R.S., Kopp S.R., Coleman G.T., Kotze A.C. (2013). Acetylcholine receptor subunit and P-glycoprotein transcription patterns in levamisole-susceptible and -resistant *Haemonchus contortus*. Int. J. Parasitol. Drugs Drug Resist..

[86] Sarai R.S., Kopp S.R., Coleman G.T., Kotze A.C. (2014). Drug-efflux and target-site gene expression patterns in *Haemonchus contortus* larvae able to survive increasing concentrations of levamisole in vitro. Int. J. Parasitol. Drugs Drug Resist..

[87] Sarai R.S., Kopp S.R., Knox M.R., Coleman G.T., Kotze A.C. (2015). In vitro levamisole selection pressure on larval stages of *Haemonchus contortus* over nine generations gives rise to drug resistance and target site gene expression changes specific to the early larval stages only. Vet. Parasitol..

[88] Rohrer S.P., Birzin E.T., Eary C.H., Schaeffer J.M., Shoop W.L. (1994). Ivermectin binding sites in sensitive and resistant *Haemonchus contortus*. J. Parasitol..

[89] Blackhall W.J., Pouliot J.F., Prichard R.K., Beech R.N. (1998). *Haemonchus contortus*: Selection at a glutamate-gated chloride channel gene in ivermectin- and moxidectin selected strains. Exp. Parasitol..

[90] McCavera S., Rogers A.T., Yates D.M., Woods D.J., Wolstenholme A.J. (2009). An ivermectin-sensitive glutamate-gated chloride channel from the parasitic nematode *Haemonchus contortus*. Mol. Pharmacol..

[91] Feng X.P., Hayashi J., Beech R.N., Prichard R.K. (2002). Study of the nematode putative GABA type-A receptor subunits: Evidence for modulation by ivermectin. J. Neurochem..

[92] Blackhall W.J., Prichard R.K., Beech R.N. (2003). Selection at a gamma-aminobutyric acid receptor gene in *Haemonchus contortus* resistant to avermectins/milbemycins. Mol. Biochem. Parasitol..

[93] Gill J.H., Lacey E. (1998). Avermectin/milbemycin resistance in trichostrongyloid nematodes. Int. J. Parasitol..

[94] Lynagh T., Lynch J.W. (2010). A glycine residue essential for high ivermectin sensitivity in Cys-loop ion channel receptors. Int. J. Parasitol..

[95] Hibbs R.E., Gouaux E. (2011). Principles of activation and permeation in an anion-selective Cys-loop receptor. Nature.

[96] Lynagh T., Lynch J.W. (2012). Ivermectin binding sites in human and invertebrate Cys-loop receptors. Trends Pharmacol. Sci..

[97] Lespine A., Ménez C., Bourguinat C., Prichard R.K. (2012). P-glycoproteins and other multidrug resistance transporters in the pharmacology of anthelmintics: Prospects for reversing transport-dependent anthelmintic resistance. Int. J. Parasitol. Drugs Drug Resist..

[98] Blackhall W.J., Liu H.Y., Xu M., Prichard R.K., Beech R.N. (1998). Selection at a P-glycoprotein gene in ivermectin and moxidectin-selected strains of *Haemonchus contortus*. Mol. Biochem. Parasitol..

[99] Xu M., Molento M., Blackhall W., Ribeiro P., Beech R., Prichard R. (1998). Ivermectin resistance in nematodes may be caused by alteration of P-glycoprotein homolog. Mol. Biochem. Parasitol..

[100] Sangster N.C., Bannan S.C., Weiss A.S., Nulf S.C., Klein R.D., Geary T.G. (1999). *Haemonchus contortus*: Sequence heterogeneity of inter-nucleotide binding domains from P-glycoproteins. Exp. Parasitol..

[101] Lloberas M., Alvarez L., Entrocasso C., Virkel G., Ballent M., Mate L., Lanusse C., Lifschitz A. (2013). Comparative tissue pharmacokinetics and efficacy of moxidectin, abamectin and ivermectin in lambs infected with resistant nematodes: Impact of drug treatments on parasite P-glycoprotein expression. Int. J. Parasitol. Drugs Drug Resist..

[102] Bartley D.J., McAllister H., Bartley Y., Dupuy J., Menez C., Alvinerie M., Jackson F., Lespine A. (2009). P-glycoprotein interfering agents potentiate ivermectin susceptibility in ivermectin sensitive and resistant isolates of *Teladorsagia circumcincta* and *Haemonchus contortus*. Parasitology.

[103] Heckler R.P., Almeida G.D., Santos L.B., Borges D.G., Neves J.P., Onizuka M.K., Borges F.A. (2014). P-gp modulating drugs greatly potentiate the in vitro effect of ivermectin against resistant larvae of *Haemonchus placei*. Vet. Parasitol..

[104] Raza A., Kopp S.R., Jabbar A., Kotze A.C. (2015). Effects of third generation P-glycoprotein inhibitors on the sensitivity of drug-resistant and -susceptible isolates of *Haemonchus contortus* to anthelmintics in vitro. Vet. Parasitol..

[105] Lifschitz A., Entrocasso C., Alvarez L., Lloberas M., Ballent M., Manazza G., Virkel G., Borda B., Lanusse C. (2010). Interference with P-glycoprotein improves ivermectin activity against adult resistant nematodes in sheep. Vet. Parasitol..

[106] Gill J.H., Kerr C.A., Shoop W.L., Lacey E. (1998). Evidence of multiple mechanisms of avermectin resistance in *Haemonchus contortus* comparison of selection protocols. Int. J. Parasitol..

[107] Rothwell J., Sangster N.C. (1997). *Haemonchus contortus*: The uptake and metabolism of closantel. Int. J. Parasitol..

[108] Kwa M.S., Okoli M.N., Schulz-Key H., Okongkwo P.O., Roos M.H. (1998). Use of P-glycoprotein gene probes to investigate anthelmintic resistance in *Haemonchus contortus* and comparison with *Onchocerca volvulus*. Int. J. Parasitol..

[109] Kaminsky R., Ducray P., Jung M., Clover R., Rufener L., Bouvier J., Weber S.S., Wenger A., Wieland-Berghausen S., Goebel T. (2008). A new class of anthelmintics effective against drug-resistant nematodes. Nature.

[110] Mederos A.E., Ramos Z., Banchero G.E. (2014). First report of monepantel *Haemonchus contortus* resistance on sheep farms in Uruguay. Parasites Vectors.

[111] Van den Brom R., Moll L., Kappert C., Vellema P. (2015). *Haemonchus contortus* resistance to monepantel in sheep. Vet. Parasitol..

[112] Bagnall N.H., Ruffell A., Raza A., Elliott T.P., Lamb J.L., Hunt P.W., Kotzea A.C. (2017). Mutations in the Hco-mptl-1 gene in a field-derived monepantel-resistant isolate of *Haemonchus contortus*. Int. J. Parasitol. Drugs Drug Resist..

[113] Van Wyk J.A., Malan F.S. (1988). Resistance of field strains of *Haemonchus contortus* to ivermectin, closantel, rafoxanide and the benzimidazoles in South Africa. Vet. Rec..

[114] Love S.C.J., Neilson F.J.A., Biddle A.J., McKinnon R. (2003). Moxidectin resistant *Haemonchus contortus* in sheep in northern New South Wales. Aust. Vet. J..

[115] Cezar A.S., Toscan G., Camillo G., Sangioni L.A., Ribas H.O., Vogel F.S. (2010). Multiple resistance of gastrointestinal nematodes to nine different drugs in a sheep flock in southern Brazil. Vet. Parasitol..

[116] Tsotetsi A.M., Njiro S., Katsande T.C., Moyo G., Baloyi F., Mpofu J. (2013). Prevalence of gastrointestinal helminths and anthelmintic resistance on small-scale farms in Gauteng Province, South Africa. Trop. Anim. Health Prod..

[117] Veríssimo C.J., Niciura S.C., Alberti A.L., Rodrigues C.F., Barbosa C.M., Chiebao D.P., Cardoso D., da Silva G.S., Pereira J.R., Margatho L.F. (2012). Multidrug and multispecies resistance in sheep flocks from Sao Paulo state, Brazil. Vet. Parasitol..

[118] Falzon L.C., Menzies P.I., Shakya K.P., Jones-Bitton A., Vanleeuwen J., Avula J., Stewart H., Jansen J.T., Taylor M.A., Learmount J. (2013). Anthelmintic resistance in sheep flocks in Ontario, Canada. Vet. Parasitol..

[119] Chandra S., Prasad A., Yadav N., Latchumikanthan A., Rakesh R.L., Praveen K., Khobra V., Subramani K.V., Misri J., Sankar M. (2015). Status of benzimidazole resistance in *Haemonchus contortus* of goats from different geographic regions of Uttar Pradesh, India. Vet. Parasitol..

[120] Playford M.C., Smith A.N., Love S., Besier R.B., Kluver P., Bailey J.N. (2014). Prevalence and severity of anthelmintic resistance in ovine gastrointestinal nematodes in Australia (2009–2012). Aust. Vet. J..

[121] Papadopoulos E., Gallidis E., Ptochos S. (2012). Anthelmintic resistance in sheep in Europe: A selected review. Vet. Parasitol..

[122] Cazajous T., Prevot F., Kerbiriou A., Milhes M., Grisez C., Tropee A., Godart C., Aragon A., Jacquiet P. (2018). Multiple resistance to ivermectin and benzimidazoles of a *Haemonchus contortus* population in a sheep flock from mainland France, first report. Vet. Parasitol. Reg. Stud. Rep..

[123] Eng J.K.L., Blackhall W.J., Osei-Atweneboana M.Y., Bourguinat C., Galazzo D., Beech R.N., Unnasch T.R., Awadzi K., Lubega G.W., Prichard R.K. (2006). Ivermectin selection on b-tubulin: Evidence in *Onchocerca volvulus* and *Haemonchus contortus*. Mol. Biochem. Parasitol..

[124] Ashraf S., Beech R.N., Hancock M.A., Prichard R.K. (2015). Ivermectin binds to *Haemonchus contortus* tubulins and promotes stability of microtubules. Int. J. Parasitol..

[125] Besier R.B., Kahn L.P., Sargison N.D., Van Wyk J.A. (2016). The pathophysiology, ecology and epidemiology of *Haemonchus contortus* infection in small ruminants. Adv. Parasitol..

[126] Levine N.D. (1980). Nematode Parasites of Domestic Animals and of Man.

[127] Veglia F., Union of South Africa, Department of Agriculture (1916). The Anatomy and Life History of the Haemonchus contortus (Rud.).

[128] Monnig H.O. (1950). Veterinary Helminthology and Entomology.

[129] Hunter A.R., McKenzie G. (1982). The pathogenesis of a single challenge dose of *Haemonchus contortus* in lambs under six months of age. J. Helminthol..

[130] Clark C.H., Kiesel G.K., Goby C.H. (1962). Measurements of blood loss caused by *Haemonchus contortus* infection in sheep. Am. J. Vet. Res..

[131] Dargie J.D., Allonby E.W. (1975). Pathophysiology of single challenge infections of *Haemonchus contortus* in Merino sheep: Studies on red cell kinetics and the “self-cure” phenomenon. Int. J. Parasitol..

[132] Albers G.A., Le Jambre L.F. (1983). Erythrocyte potassium concentration: A simple parameter for erythropoiesis in sheep infected with *Haemonchus contortus*. Res. Vet. Sci..

[133] Le Jambre L.F. (1995). Relationship of blood loss to worm numbers, biomass and egg production in *Haemonchus contortus* infected sheep. Int. J. Parasitol..

[134] Dunn A.M. (1978). Veterinary Helminthology.

[135] Suarez V.H., Cristel S.L., Busetti M.R. (2009). Epidemiology and effects of gastrointestinal nematode infection on milk productions of dairy ewes. Parasite.

[136] Albers G.A.A., Gray G.D., Le Jambre L.F., Piper L.R., Barger I.A., Barker J.S.F. (1989). The effect of *Haemonchus contortus* on liveweight gain and wool growth in young Merino sheep. Aust. J. Agric. Res..

[137] Van Wyk J.A. (2008). Production trials involving use of the FAMACHA© system for haemonchosis in sheep: Preliminary results. Onderstepoort J. Vet. Res..

[138] Cobon D.H., O’Sullivan B.M. (1992). Effect of *Haemonchus contortus* on productivity of ewes, lambs and weaners in a semi-arid environment. J. Agric. Sci..

[139] Barger I.A., Cox G.W. (1984). Wool production in sheep chronically infected with *Haemonchus contortus*. Vet. Parasitol..

[140] Beriajaya, Copeman D.B. (2006). *Haemonchus contortus* and *Trichostrongylus colubriformis* in pen-trials with Javanese thin tail sheep and Kacang cross Etawah goats. Vet. Parasitol..

[141] Howlader M.M.R., Capitan S.S., Eduardo S.L., Roxas N.P., Sevilla C.C. (1997). Performance of growing goats experimentally infected with stomach worm (*Haemonchus contortus*). Asian Australas. J. Anim..

[142] Githigia S.M., Thamsborg S.M., Munyua W.K., Maingi N. (2001). Impact of gastrointestinal helminths on production in goats in Kenya. Small Rumin. Res..

[143] Malan F.S., Van Wyk J.A., Wessels C.D. (2001). Clinical evaluation of anaemia in sheep: Early trials. Onderstepoort J. Vet. Res..

[144] Van Wyk J.A., Bath G.F. (2002). The FAMACHA© system for managing haemonchosis in sheep and goats by clinically identifying individual animals for treatment. Vet. Res..

[145] Moors E., Gauly M. (2009). Is the FAMACHA© chart suitable for every breed? Correlations between FAMACHA© scores and different traits of mucosa colour in naturally parasite infected breeds. Vet. Parasitol..

[146] Papadopoulos E., Gallidis E., Ptochos S., Fthenakis G.C. (2013). Evaluation of the FAMACHA© system for targeted selective anthelmintic treatments for potential use in small ruminants in Greece. Small Rumin. Res..

[147] Van Wyk J.A., Mayhew E. (2013). Morphological identification of parasitic nematode infective larvae of small ruminants and cattle: A practical lab guide. Onderstepoort J. Vet. Res..

[148] Roberts J.L., Swan R.A. (1982). Quantitative studies of ovine haemonchosis. Relationship between total worm counts of *Haemonchus contortus*, haemoglobin values and bodyweight. Vet. Parasitol..

[149] Beh K.J., Foley R.C., Goodwin E.J. (1989). Restriction fragment length patterns of DNA from parasitic nematodes of sheep. Res. Vet. Sci..

[150] Christensen C.M., Zarlenga D.S., Gasbarre L.C. (1994). *Ostertagia, Haemonchus, Cooperia*, and *Oesophagostomum*: Construction and characterization of genus-specific DNA probes to differentiate important parasites of cattle. Exp. Parasitol..

[151] Christensen C.M., Zarlenga D.S., Gasbarre L.C. (1994). Identification of a *Haemonchus placei*-specific DNA probe. J. Helminthol. Soc. Wash..

[152] Stevenson L.A., Chilton N.B., Gasser R.B. (1995). Differentiation of *Haemonchus placei* from *H. contortus* (Nematoda: Trichostrongylidae) by the ribosomal DNA second internal transcribed spacer. Int. J. Parasitol..

[153] Troell K., Engstrom A., Morrison D.A., Mattsson J.G., Hoglund J. (2006). Global patterns reveal strong population structure in *Haemonchus contortus*, a nematode parasite of domesticated ruminants. Int. J. Parasitol..

[154] Chaudhry U., Redman E.M., Abbas M., Muthusamy R., Ashraf K., Gilleard J.S. (2015). Genetic evidence for hybridisation between *Haemonchus contortus* and *Haemonchus placei* in natural field populations and its implications for interspecies transmission of anthelmintic resistance. Int. J. Parasitol..

[155] Roos M.H., Boersema J.H., Borgsteede F.H., Cornelissen J., Taylor M., Ruitenberg E.J. (1990). Molecular analysis of selection for benzimidazole resistance in the sheep parasite *Haemonchus contortus*. Mol. Biochem. Parasitol..

[156] Zarlenga D.S., Stringfellow F., Nobary M., Lichtenfels J.R. (1994). Cloning and characterization of ribosomal RNA genes from three species of *Haemonchus* (Nematoda: Trichostrongyloidea) and identification of PCR primers for rapid differentiation. Exp. Parasitol..

[157] Roos M.H., Grant W.N. (1993). Species-specific PCR for the parasitic nematodes *Haemonchus contortus* and *Trichostrongylus colubriformis*. Int. J. Parasitol..

[158] Heise M., Epe C., Schnieder T. (1999). Differences in the second internal transcribed spacer (ITS-2) of eight species of gastrointestinal nematodes of ruminants. J. Parasitol..

[159] Pichler R., Silbermayr K., Periasamy K. (2017). A novel snapback primer probe assay for the detection and discrimination of sympatric *Haemonchus* species using DNA melting analysis. Vet. Parasitol..

[160] Blouin M.S., Yowell C.A., Courtney C.H., Dame J.B. (1997). *Haemonchus placei* and *Haemonchus contortus* are distinct species based on mtDNA evidence. Int. J. Parasitol..

[161] Blouin M.S., Yowell C.A., Courtney C.H., Dame J.B. (1998). Substitution bias, rapid saturation and the use of mtDNA for nematode systematics. Mol. Biol. Evol..

[162] Roeber F., Jex A.R., Campbell A.J., Nielsen R., Anderson G.A., Stanley K.K., Gasser R.B. (2012). Establishment of a robotic, high-throughput platform for the specific diagnosis of gastrointestinal nematode infections in sheep. Int. J. Parasitol..

[163] Demeler J., Ramunke S., Wolken S., Ianiello D., Rinaldi L., Gahutu J.B., Cringoli G., von Samson-Himmelstjerna G., Krucken J. (2013). Discrimination of gastrointestinal nematode eggs from crude fecal egg preparations by inhibitor resistant conventional and real-time PCR. PLoS ONE.

[164] Von Samson-Himmelstjerna G., Harder A., Schnieder T. (2002). Quantitative analysis of ITS2 sequences in trichostrongyle parasites. Int. J. Parasitol..

[165] Siedek E.M., Burden D., von Samson-Himmelstjerna G. (2006). Feasibility of genus-specific real-time PCR for the differentiation of larvae from gastrointestinal nematodes of naturally infected sheep. Berl. Munch. Tierarztl. Wochenschr..

[166] Harmon A.F., Williams Z.B., Zarlenga D.S., Hildreth M.B. (2007). Real-time PCR for quantifying *Haemonchus contortus* eggs and potential limiting factors. Parasitol. Res..

[167] Learmount J., Conyers C., Hird H., Morgan C., Craig B.H., von Samson-Himmelstjerna G., Taylor M. (2009). Development and validation of real-time PCR methods for diagnosis of *Teladorsagia circumcincta* and *Haemonchus contortus* in sheep. Vet. Parasitol..

[168] McNally J., Callan D., Andronicos N., Bott N., Hunt P.W. (2013). DNA-based methodology for the quantification of gastrointestinal nematode eggs in sheep faeces. Vet. Parasitol..

[169] Roeber F., Jex A.R., Gasser R.B. (2013). Advances in the diagnosis of key gastrointestinal nematode infections of livestock, with an emphasis on small ruminants. Biotechnol. Adv..

[170] Roeber F., Jex A.R., Gasser R.B. (2013). Next-generation molecular-diagnostic tools for gastrointestinal nematodes of livestock, with an emphasis on small ruminants: A turning point?. Adv. Parasitol..

[171] Preston S.J., Sandeman M., Gonzalez J., Piedrafita D. (2014). Current status for gastrointestinal nematode diagnosis in small ruminants: Where are we and where are we going?. J. Immunol. Res..

[172] Sweeny J.P., Robertson I.D., Ryan U.M., Jacobson C., Woodgate R.G. (2011). Comparison of molecular and McMaster microscopy techniques to confirm the presence of naturally acquired strongylid nematode infections in sheep. Mol. Biochem. Parasitol..

[173] Roeber F., Larsen J.W., Anderson N., Campbell A.J., Anderson G.A., Gasser R.B., Jex A.R. (2012). A molecular diagnostic tool to replace larval culture in conventional faecal egg count reduction testing in sheep. PLoS ONE.

[174] Elmahalawy S.T., Halvarsson P., Skarin M., Höglund J. (2018). Droplet digital polymerase chain reaction (ddPCR) as a novel method for absolute quantification of major gastrointestinal nematodes in sheep. Vet. Parasitol..

[175] Kaminsky R., Gauvry N., Schorderet Weber S., Skripsky T., Bouvier J., Wenger A., Schroeder F., Desaules Y., Hotz R.T., Hosking B.C. (2008). Identification of the amino-acetonitrile derivative monepantel (AAD 1566) as a new anthelmintic drug development candidate. Parasitol. Res..

[176] Sager H., Hosking B., Bapst B., Stein P., Vanhoff K., Kaminsky R. (2009). Efficacy of the amino-acetonitrile derivative, monepantel, against experimental and natural adult stage gastrointestinal nematode infections in sheep. Vet. Parasitol..

[177] Besier R.B., Love S.C.J. (2003). Anthelmintic resistance in sheep nematodes in Australia: The need for new approaches. Aust. J. Exp. Agri..

[178] Cringoli G., Veneziano V., Jackson F., Vercruysse J., Greer A.W., Fedele V., Mezzino L., Rinaldi L. (2008). Effects of strategic anthelmintic treatments on the milk production of dairy sheep naturally infected by gastrointestinal strongyles. Vet. Parasitol..

[179] Van Wyk J.A., Hoste H., Kaplan R.M., Besier R.B. (2006). Targeted selective treatment for worm management-how do we sell rational programs to farmers?. Vet. Parasitol..

[180] Martin P.J., Le Jambre L.F., Claxton J.H. (1981). The impact of refugia on the development of thiabendazole resistance in *Haemonchus contortus*. Int. J. Parasitol..

[181] Torres-Acosta J.F., Hoste H. (2008). Alternative or improved methods to limit gastrointestinal parasitism in grazing/browsing sheep and goats. Small Rumin. Res..

[182] Hoste H., Le Frileux Y., Pommaret A. (2001). Distribution and repeatability of faecal egg counts and blood parameters in dairy goats naturally infected with gastrointestinal nematodes. Res. Vet. Sci..

[183] Gaba S., Ginot V., Cabaret J. (2005). Modelling macroparasite aggregation using a nematode-sheep system: The Weibull distribution as an alternative to the negative binomial distribution?. Parasitology.

[184] Sréter T., Molnár V., Kassai T. (1994). The distribution of nematode egg counts and larval counts in grazing sheep and their implications for parasite control. Int. J. Parasitol..

[185] Cabaret J. (2008). Pros and cons of targeted selective treatment against digestive-tract strongyles of ruminants. Parasite.

[186] Cabaret J. (2004). Efficacy evaluation of anthelmintics: Which methods to use in the field?. Parasitologia.

[187] Stafford K.A., Morgan E.R., Coles G.C. (2009). Weight-based targeted selective treatment of gastrointestinal nematodes in a commercial sheep flock. Vet. Parasitol..

[188] Hoste H., Le Frileux Y., Pommaret A. (2002). Comparison of selective and systematic treatments to control nematode infection of the digestive tract in dairy goats. Vet. Parasitol..

[189] Leathwick D.M., Waghorn T.S., Miller C.M., Candy P.M., Oliver A.M. (2012). Managing anthelmintic resistance—Use of a combination anthelmintic and leaving some lambs untreated to slow the development of resistance to ivermectin. Vet. Parasitol..

[190] Cornelius M.P., Jacobson C., Dobson R., Besier R.B. (2016). Computer modelling of anthelmintic resistance and worm control outcomes for refugia-based nematode control strategies in Merino ewes in Western Australia. Vet. Parasitol..

[191] Bairden K., Armour J., Duncan J.L. (1995). A 4-year study on the effectiveness of alternate grazing of cattle and sheep in the control of bovine parasitic gastroenteritis. Vet. Parasitol..

[192] Hoste H., Guitard J.P., Pons J.C. (2003). Paturage mixte entre ovins et bovines intereκt dans la gestion des strongyloses gastro intestinales. Fourrages.

[193] Sayers G., Sweeney T. (2005). Gastrointestinal nematode infection in sheep-a review of the alternatives to anthelmintics in parasite control. Anim. Health Res. Rev..

[194] Marley C.L., Fraser M.D., Davies D.A., Rees M.E., Vale J.E., Forbes A.B. (2006). The effect of mixed or sequential grazing of cattle and sheep on the faecal egg counts and growth rates of weaned lambs when treated with anthelmintics. Vet. Parasitol..

[195] Kyriazakis I., Houdijk J. (2006). Immunonutrition: Nutritional control of parasites. Small Rumin. Res..

[196] Strain S.A., Stear M.J. (2001). The influence of protein supplementation on the immune response to *Haemonchus contortus*. Parasitol. Immunol..

[197] Eady S.J., Woolaston R.R., Barger I.A. (2003). Comparison of genetic and nongenetic strategies for control of gastrointestinal nematodes of sheep. Livest. Prod. Sci..

[198] Kahn L.P., Knox M., Gray G. (2003). Enhancing immunity to nematode parasites in single-bearing Merino ewes through nutrition and genetic selection. Vet. Parasitol..

[199] Flores-Crespo J., Herrera-Rodríguez D., Mendoza de Gives P., Liébano-Hernández E., Vázquez-Prats V.M., López-Arellano M.E. (2004). The predatory capability of three nematophagous fungi in the control of *Haemonchus contortus* infective larvae in ovine faeces. J. Helminthol..

[200] Waller P.J., Rydzik A., Ljungström B.L., Törnquist M. (2004). Towards the eradication of *Haemonchus contortus* from sheep flocks in Sweden. Vet. Parasitol..

[201] Athanasiadou S., Kyriazakis I., Jackson F., Coop R.L. (2001). Direct anthelmintic effects of condensed tannins towards different gastrointestinal nematodes of sheep: In vitro and in vivo studies. Vet. Parasitol..

[202] Butter N.L., Dawson J.M., Wakelin D., Buttery P.J. (2001). Effect of dietary condensed tannins on gastrointestinal nematodes. J. Agric. Sci..

[203] Paolini V., Dorchies P., Hoste H. (2003). Effects of sainfoin hay on gastrointestinal nematode infections in goats. Vet. Rec..

[204] Paolini V., De La Farge F., Prevot F., Dorchies P., Hoste H. (2005). Effects of the repeated distribution of sainfoin hay on the resistance and the resilience of goats naturally infected with gastrointestinal nematodes. Vet. Parasitol..

[205] Mahieu M., Aumont G. (2009). Effects of sheep and cattle alternate grazing on sheep parasitism and production. Trop. Anim. Health Prod..

[206] Ruiz-Huidobro C., Sagot L., Lugagne S., Huang Y., Milhes M., Bordes L., Prévot F., Grisez C., Gautier D., Valadier C. (2019). Cell grazing and *Haemonchus contortus* control in sheep: Lessons from a two-year study in temperate Western Europe. Scient. Rep..

[207] Colvin A.F., Walkden-Brown S.W., Knox M.R., Scott M.J. (2008). Intensive rotational grazing assists control of gastrointestinal nematodosis of sheep in a cool temperate environment with summer-dominant rainfall. Vet. Parasitol..

[208] Steel J.W. (2003). Effects of protein supplementation on young sheep on resistance development and resilience to parasitic nematodes. Aust. J. Exp. Agric..

[209] Coop R.L., Holmes P.H. (1996). Nutrition and parasite interaction. Int. J. Parasitol..

[210] Abbott E.M., Parkins J.J., Holmes P.H. (1986). The effect of dietary protein on the pathogenesis of acute ovine haemonchosis. Vet. Parasitol..

[211] Wallace D.S., Bairden K., Duncan J.L., Fishwick G., Gill M., Holmes P.H., McKellar Q.A., Murray M., Parkins J.J., Stear M. (1996). Influence of soyabean meal supplementation on the resistance of Scottish Blackface lambs to haemonchosis. Res. Vet. Sci..

[212] Nnadi P.A., Kamalu T.N., Onah D.N. (2009). The effect of dietary protein on the productivity of West African Dwarf (WAD) goats infected with *Haemonchus contortus*. Vet. Parasitol..

[213] Houdijk J.G.M., Kyriazakis I., Kidanea A., Athanasiadou S. (2012). Manipulating small ruminant parasite epidemiology through the combination of nutritional strategies. Vet. Parasitol..

[214] Torres-Acosta J.F.J., Sandoval-Castro C.A., Hoste H., Aguilar-Caballero A.J., Camara-Sarmiento R., Alonso-Díaz M.A. (2012). Nutritional manipulation of sheep and goats for the control of gastrointestinal nematodes under hot humid and subhumid tropical conditions. Small Rumin. Res..

[215] Hoste H., Martínez-Ortiz-De-Montellano C., Manolaraki F., Brunet S., Ojeda-Robertos N., Fourquaux I., Torres-Acosta J.F.J., Sandoval-Castro C.A. (2012). Direct and indirect effects of bioactive tannin-rich tropical and temperate legumes against nematode infections. Vet. Parasitol..

[216] Athanasiadou S., Tzamaloukas O., Kyriazakis I., Jackson F., Coop R.L. (2005). Testing for direct anthelmintic effects of bioactive forages against *Trichostrongylus colubriformis* in grazing sheep. Vet. Parasitol..

[217] Waghorn G. (2008). Beneficial and detrimental effects of dietary condensed tannins for sustainable sheep and goat production—Progress and challenges. Anim. Feed Sci. Technol..

[218] Waller P.J., Larsen M. (1993). The role of nematophagous fungi in the biological control of nematode parasites of livestock. Int. J. Parasitol..

[219] Kelly P., Good B., Hanrahan J.P., Fitzpatrick R., de Waal T. (2009). Screening for the presence of nematophagous fungi collected from Irish sheep pastures. Vet. Parasitol..

[220] Waller P.J., Knox M.R., Faedo M. (2009). The potential of nematophagous fungi to control the free-living stages of nematodes of sheep: Feeding and block studies with *Duddingtonia flagrans*. Vet. Parasitol..

[221] Chandrawathani P., Jamnah O., Adnan M., Waller P.J., Larsen M., Gillespie A.T. (2004). Field studies on the biological control of nematode parasites of sheep in the tropics, using the microfungus *Duddingtonia flagrans*. Vet. Parasitol..

[222] Maingi N., Krecek R.C., Van Biljon N. (2006). Control of gastrointestinal nematodes in goats on pastures in South Africa using nematophagous fungi *Duddingtonia flagrans* and selective anthelmintic treatments. Vet. Parasitol..

[223] Mugambi J.M., Bain R.K., Wanyangu S.W., Ihiga M.A., Duncan J.L., Murray M., Stear M.J. (1997). Resistance of four sheep breeds to natural and subsequent artificial *Haemonchus contortus* infection. Vet. Parasitol..

[224] Terefe G., Lacroux C., Andreoletti O., Grisez C., Prevot F., Bergeaud J.P., Penicaud J., Rouillon V., Gruner L., Brunel J.C. (2007). Immune response to Haemonchus contortus infection in susceptible (INRA 401) and resistant (Barbados Black Belly) breeds of lambs. Parasite Immunol..

[225] Benavides M.V., Sonstegard T.S., Kemp S., Mugambi J.M., Gibson J.P., Baker R.L., Hanotte O., Marshall K., Van Tassell C. (2015). Identification of novel loci associated with gastrointestinal parasite resistance in a Red Maasai x Dorper backcross population. PLoS ONE.

[226] Barbosa Toscano J.H., dos Santos I.B., von Haehling M.B., Giraldelo L.A., Lopes L.G., da Silva M.H., Figueiredo A., Esteves S.N., Souza Chagas A.C. (2019). Morada Nova sheep breed: Resistant or resilient to *Haemonchus contortus* infection?. Vet. Parasitol. X.

[227] Estrada-Reyes Z.M., Tsukahara Y., Amadeu R.R., Goetsch A.L., Gipson T.A., Sahlu T., Puchala R., Wang Z., Hart S.P., Mateescu R.G. (2019). Signatures of selection for resistance to *Haemonchus contortus* in sheep and goats. BMC Genom..

[228] Woolaston R.R., Baker R.L. (1996). Prospects of breeding small ruminants for resistance to internal parasites. Int. J. Parasitol..

[229] Aguerre S., Jacquiet P., Brodier H., Bournazel J.P., Grisez C., Prévot F., Michot L., Fidelle F., Astruc J.M., Moreno C.R. (2018). Resistance to gastrointestinal nematodes in dairy sheep: Genetic variability and relevance of artificial infection of nucleus rams to select for resistant ewes on farms. Vet. Parasitol..

[230] Becker G., Davenport K., Burke J.M., Lewis R.M., Miller J., Morgan J., Notter D., Murdoch B. (2020). Genome-wide association study to identify genetic loci associated with gastrointestinal nematode resistance in Katahdin sheep. Anim. Gen..

[231] Kelly G.A., Kahn L.P., Walkden-Brown S.W. (2013). Measurement of phenotypic resilience to gastro-intestinal nematodes in Merino sheep and association with resistance and production variables. Vet. Parasitol..

[232] Emery D.L., Hunt P.W., Le Jambre L.F. (2016). *Haemonchus contortus*: The then and now, and where to from here?. Int. J. Parasitol..

[233] Nisbet A.J., Meeusen E.N., Gonzalez J.F., Piedrafita D.M. (2016). Immunity to *Haemonchus contortus* and vaccine development. Adv. Parasitol..

[234] Tian X., Ly M., Jia C., Bu Y., Aimulajiang K., Zhang Y., Li C., Yan R., Xu L., Song X. (2020). *Haemonchus contortus* transthyretin domain containing protein (HcTTR): A promising vaccine candidate against *Haemonchus contortus* infection. Vet. Parasitol..

[235] Andrews S.J., Rolph T.P., Munn E.A. (1997). Duration of protective immunity against ovine haemonchosis following vaccination with the nematode gut membrane antigen H11. Res. Vet. Sci..

[236] Bassetto C.C., Picharillo M.E., Newlands G.F.J., Smith W.D., Fernandes S., Siqueira E.R., Amarante A.F.T. (2014). Attempts to vaccinate ewes and their lambs against natural infection with *Haemonchus contortus* in a tropical environment. Int. J. Parasitol..

[237] Besier R.B., Smith W.D. A new approach to the control of barbers poe worm. Proceedings of the 2014 Conference of Australian Sheep Veterinarians.

[238] Morgan E.R., Aziz N.A.A., Blanchard A., Charlier J., Charvet C., Claerebout E., Geldhof P., Greer A.W., Hertzberg H., Hodgkinson J. (2019). 100 Questions in livestock helminthology research. Trends Parasitol..

[239] Hoberg E.P., Lichtenfels J.R., Gibbons L.M. (2004). Phylogeny for species of *Haemonchus* (Nematoda: Trichostrongyloidea): Considerations of their evolutionary history and global biogeography among Camelidae and Pecora (Artiodactyla). J. Parasitol..

